# Synthetic control methodology as a tool for evaluating population-level health interventions

**DOI:** 10.1136/jech-2017-210106

**Published:** 2018-04-13

**Authors:** Janet Bouttell, Peter Craig, James Lewsey, Mark Robinson, Frank Popham

**Affiliations:** 1 Health Economics and Health Technology Assessment, Institute of Health and Wellbeing, University of Glasgow, Glasgow, UK; 2 MRC/CSO Social and Public Health Sciences Unit, Institute of Health and Wellbeing, University of Glasgow, Glasgow, UK; 3 Public Health Observatory, NHS Health Scotland, Glasgow, UK

**Keywords:** public health, health impact assessment, methodology, research methods

## Abstract

**Background:**

Many public health interventions cannot be evaluated using randomised controlled trials so they rely on the assessment of observational data. Techniques for evaluating public health interventions using observational data include interrupted time series analysis, panel data regression-based approaches, regression discontinuity and instrumental variable approaches. The inclusion of a counterfactual improves causal inference for approaches based on time series analysis, but the selection of a suitable counterfactual or control area can be problematic. The synthetic control method builds a counterfactual using a weighted combination of potential control units.

**Methods:**

We explain the synthetic control method, summarise its use in health research to date, set out its advantages, assumptions and limitations and describe its implementation through a case study of life expectancy following German reunification.

**Results:**

Advantages of the synthetic control method are that it offers an approach suitable when there is a small number of treated units and control units and it does not rely on parallel preimplementation trends like difference in difference methods. The credibility of the result relies on achieving a good preimplementation fit for the outcome of interest between treated unit and synthetic control. If a good preimplementation fit is established over an extended period of time, a discrepancy in the outcome variable following the intervention can be interpreted as an intervention effect. It is critical that the synthetic control is built from a pool of potential controls that are similar to the treated unit. There is currently no consensus on what constitutes a ‘good fit’ or how to judge similarity. Traditional statistical inference is not appropriate with this approach, although alternatives are available. From our review, we noted that the synthetic control method has been underused in public health.

**Conclusions:**

Synthetic control methods are a valuable addition to the range of approaches for evaluating public health interventions when randomisation is impractical. They deserve to be more widely applied, ideally in combination with other methods so that the dependence of findings on particular assumptions can be assessed.

## Background and introduction

Population-level health interventions aim to improve health by changing underlying social, economic and environmental conditions, or by directly influencing health behaviours. They may be specific to healthcare or involve the regulation of or changes to sectors such as education, transport, housing or employment. Often, randomised approaches to evaluation are not possible for population-level health interventions for practical, political or ethical reasons, for example, where a smoking ban is introduced across the entire population of a country. Non-randomised approaches using observational data are, therefore, the only methods left open to evaluators. Providing that suitable methods have been adopted to ensure the internal validity of the study, observational studies have much to contribute.

In order to minimise bias (ie, the risk that the effect seen is due to factors other than the intervention), many studies use a control group or counterfactual. In establishing a counterfactual, different study designs use different methods to mimic the trend in the outcome variable in the absence of the intervention. Some methods, such as direct matching and propensity score matching, select controls based only on observed characteristics. Regression discontinuity analysis uses individuals on the opposite side of a cut-off as the control group, while the instrumental variable technique uses a variable that influences the explanatory variable but not the dependent variable, thereby allowing a researcher to separate the intervention effect. Other approaches, such as uncontrolled ‘before and after’ approaches, rely on the assumption that the previous trend in the treated unit would have continued but for the intervention only. Here, the modelled continuation of the pretrend is the counterfactual. When a control unit is added, the change in the treated unit is compared with the change in the control in what is known as a ‘difference in difference’ (DiD) approach. DiD estimates the treatment effect by taking the difference between the change in the treated unit and the change in the control units in the periods before and after the intervention. Provided that treated and control units had parallel pretreatment trends, and there were no other events affecting one unit but not the other, a constant difference would be expected to continue in the postintervention period. If the difference between the treated and control unit changes in the postintervention period, this can be interpreted as a treatment effect. If pretreatment trends are not parallel, or an event occurred that affected only one of the units, some of the difference between the units will be the difference in the trend rather than the effect of the intervention, and the estimate of the treatment effect will be biased.

It is sometimes difficult to establish whether the parallel trends assumption is met and whether the control group is a sufficiently accurate representation of what would have happened in the treated area without the intervention. Synthetic control methodology (SCM) allows the construction of a counterfactual by selecting a weighted average of the outcome variable from a group of units similar to the treated unit.

Abadie and Gardeazabal^1^ first set out SCM in a study looking at the impact of terrorism on the economy of the Basque region of Spain. Abadie *et al* 2 followed this with a study examining the impact of California’s 1988 tobacco control programme, and statistical packages (in Stata, R and Matlab) to support the implementation of the method were released in 2011. A further overview of the method and case study looking at the economic impact on West Germany of reunification in 1990^3^ was published in 2015 and is a useful source of reference.

The aims of this article are to introduce SCM to researchers in public health, to set out the uses of SCM in health as identified by our review and to provide a step-by-step guide to implementation and case study with syntax and data (adapted from Abadie *et al* 3).

## Method

We undertook a wide-ranging literature review in February 2016 searching the term ‘synthetic control method’ or ‘synthetic control’ in 26 health, social science and grey literature databases (see online supplementary material 1). Key authors and citations of key articles were also searched. No restrictions were placed on language or date. The only inclusion criterion was that the article described an application of SCM.

10.1136/jech-2017-210106.supp1 Supplementary file 1

## Results

First, we summarise examples of the use of SCM in health research as identified in our review, we then set out the steps in conducting an SCM study and outline the advantages, assumptions and limitations of the method. In online supplementary material 2, we present a further case study of German reunification, this time looking at its impact on life expectancy, we illustrate the method set out in those articles in straightforward steps. We use Stata V.14 and synth for Stata. Data and syntax are provided in online supplementary material 3.

10.1136/jech-2017-210106.supp2 Supplementary file 2

### Use of SCM in health

Table 1 sets out the exposures and outcomes, settings and study level of 38 health-related studies using the synthetic control method identified in the literature review^2 4–39^ (and the unpublished Mas N, Friedman J and Figallo M. Can integrated primary and hospital care improve both quality and efficiency outcomes in health care? Evidence from a Spanish Public private partnership (2015) (permission to quote granted)).

**Table 1:** Health-related studies using synthetic control methodology

Date	First author (reference)	Exposure	Outcome	Treated unit(s)	Donor pool
Health finance and health systems reform					
2016	Ryan^4^	Pay for performance	Mortality	UK	Other high-income countries
2015	Mas (unpublished)	Healthcare integration	Healthcare efficiency measures	Spanish integrated healthcare unit	Spanish non-integrated healthcare units
2015	Lepine^5^	User fee removal	Healthcare utilisation	Treated Zambian regions	Untreated Zambian regions
2015	Kreif^6^	Pay for performance	Risk adjusted mortality	Treated UK hospitals	Untreated UK hospitals
2015	Machado^7^	Levels of health insurance	Infertility levels	States with strong infertility mandates	States with weak infertility mandates
2015	Roy^8^	Health reform	Sources of health insurance	Insured Massachusetts Population	Uninsured Massachusetts population
2014	Courtemanche^9^	Levels of health insurance	Self-reported health	Massachusetts	Untreated US states
2014	Dunn^10^	Health reform	Physician payments	Massachusetts	Untreated US states
2014	Tuzemen^11^	Health reform	Non-insurance rate	Massachusetts	Untreated US states
2013	Lo^12^	Income levels	Substitution of public/private insurance	Illinois	Untreated US states
Industry regulation					
2016	Quast^13^	Registration of sex workers	Incidence of sexually transmitted disease	Tijuana, Mexico	Untreated Mexican regions
2015	Restrepo^14^	Trans fat ban	Heart disease mortality	Treated counties in New York	Untreated counties in New York
2014	Sampaio^15^	Mobile phone ban	Road accidents	New York state	Untreated US states
2014	Restrepo^16^	Trans fat ban	Heart disease mortality	Denmark	Other OECD countries
2014	Green^17^	Alcohol licencing hours	Road accidents	England and Wales	Scottish regions
2014	Wang^18^	Pasteurisation of milk	Child mortality	Treated US cities	Untreated US cities
2014	Cunningham^19^	Decriminalisation of indoor prostitution	Incidence of sexually transmitted disease	Rhode Island, USA	Untreated US states/cities
Taxation policy					
2016	Berardi^20^	Tax on sugary drinks	Price of affected drink categories	Prices of sugary drinks in France	Other drink categories in France
2016	Grogger^21^	Tax on sugary drinks	Price of other drink categories	Prices of sugary drinks in Mexico	Other drink categories in Mexico
2016	Chelwa^22^	Tax on cigarettes	Cigarette sales	South Africa	Untreated low-income and middle-income countries
2015	Bilgel^23^	Tax breaks for organ donors	Live organ donations	New York State	Untreated US states
2015	Fletcher^24^	Tax on sugary drinks	Changes in BMI	Arkansas, Ohio	Untreated US states
2010	Abadie^2^	Tax on cigarettes	Cigarette sales	California	Untreated US states
Legal changes					
2015	Zabinski^25^	Medical malpractice liability	Patient safety measures	Texas, Florida, Illinois, South Carolina, Georgia	Untreated US states
2015	Crifasi^26^	Hand gun laws	Suicide rates	Connecticut, Missouri	Untreated US states
2015	Cunningham^27^	Drug laws	Drug seizures	Mississippi	Untreated US states
2015	Powell^28^	Legalisation of marijuana	Overdoses from painkillers	US states with medical marijuana law	US states without medical marijuana law
Development and barriers to development					
2015	Alavuotunki^29^	General Budget Support (GBS)	Neonatal mortality	Countries in receipt of GBS	Countries from the region of the treated country not in receipt of GBS
2015	Karlsson^30^	HIV prevalence	Demographic outcomes	Countries in Africa with high HIV prevalence	Countries in Africa with low HIV prevalence
2014	Pereda^31^	Climate change	Dengue risk	Brazilian cities with high dengue risk	Brazilian cities with low dengue risk
2014	Peiters^32^	Political transition	Child mortality	Countries which became democratic	Countries that remained autocratic
Nutrition interventions					
2015	Qian^33^	School food programme	BMI	Treated schools in Arkansas, US	Untreated schools in Arkansas, USA
2014	Bauhoff^34^	School food programme	BMI	Treated school districts in California, US	Untreated school district in California, USA
2013	Kiesel^35^	Better food labelling	Product sales	Treated US supermarkets	Untreated US supermarkets
Welfare reforms					
2016	Chung^36^	Money transfer	Birth weights	Alaska	Untreated US states
2016	Oloomi^37^	Paid family leave	Infant health outcomes	California	Untreated US states
2016	Basu^38^	Welfare reforms	Healthcare utilisation	Single mothers in the USA	Married mothers and single women in the USA
2015	Grossman^39^	Urban empowerment zones (UEZs)	Fertility and mental health outcomes	UEZ in Chicago, New York and Philadephia	Unsuccessful applicants for UEZ status in US cities

BMI, body mass index; OECD, Organisation for Economic Co-operation and Development.

The majority of the studies examined the impact of interventions imposed at a US state or national level, such as welfare and health system reforms, legislation, taxation or industry regulation, for which randomisation would be impractical. A few studies sought to identify the effects of other population-wide exposures such as climate or political regime change. Most studies focused on a single ‘treated unit’ where the change took place, and SCM was used in order to select a counterfactual with a good preimplementation fit for the single unit. We cannot be sure we have identified all studies using synthetic control methods particularly if they have used alternative terminology (ie, not ‘synthetic control’) and have not referred to the Abadie *et al* papers.^1–3^

### Steps in conducting an SCM study

The basic steps of the approach are:

1. Ensure the theory behind the intervention is well understood. Develop or present a conceptual model to make the theory transparent. This allows appropriate independent variables and possible confounding variables (collectively referred to hereafter as ‘predictor variables’ in line with Abadie *et al* 1–3 to be included in the analysis. It also allows researchers to ensure areas that have also been exposed to a similar intervention are excluded from the pool of potential controls (the ‘donor pool’) (see table 2 for key assumptions of SCM).
2. Identify potential control units. It is essential for the credibility of the method that the donor pool only contains units that are similar to the treated unit in aspects important to the outcome (see box 1).
3. Develop the synthetic control. An optimisation procedure using the outcome variables from the potential control areas and any other predictor variables identified (see box 2) selects the best weighting of units from the donor pool to create a synthetic control. The optimisation procedure minimises the difference between the outcome of interest in the treated unit in the preintervention period and the synthetic control. The difference is measured by the root mean square prediction error (RMSPE).
4. Run outcome analysis. Once the composition of the synthetic control has been established using only preintervention data, the postintervention data can be added and the outcome analysis run.
5. Present results. If the intervention has had an effect this should be visible from a graph comparing the postintervention outcome with the weighted control outcome (ie, the ‘synthetic control’ outcome).
6. Run robustness checks. Placebo analysis can be used as a falsification test as traditional statistical inference is inappropriate in situations where there are small numbers of treated and control units and because units are not sampled probabilistically. Placebo analysis involves performing the analysis as if other units in the donor pool were the treated unit to generate a distribution of effect estimates. If the intervention is the cause of the observed effect, then the gap between the treated and its synthetic control outcome should be largest for the actual treated unit.

**Table 2:** Key assumptions of synthetic control methodology

Assumption	Assessment
1. Treated units and potential control units in the donor pool are similar.	Similar levels in variables known to influence outcome variable (see box 1 for objective and subjective elements of this assessment).
2. There is no contamination – spillover of effects of intervention into potential control units.	Based on background knowledge of researchers.
3. No external shocks in potential control units.	Based on background knowledge of researchers informed by review of trends in outcome variable.

A further useful step is to compare the synthetic control-based estimates to effect estimates obtained using other methods. A number of published SCM studies make such comparisons, most often with DiD methods^6 7 26 39^ and also with pre and post approaches,^8 10^ propensity score matching^5^ and lagged dependent variable regression.^40^ Ideally, the comparisons should be prespecified, and the likely biases of each approach made explicit.

Box 1 **Similarity of the treated and potential control units**

In assessing the *similarity* of the potential control units there are subjective and objective aspects.

The subjective aspects are:

- Although the choice of predictor variables should be driven by existing theory or a conceptual model, there remains subjectivity in the choice of time periods to include.
- The choice of the donor pool (whether it is appropriate to include, eg, all countries of the world, all states of the USA, just economically developed countries, countries of a similar population size and so on).
- The limits of predictor variables that would indicate whether a potential control unit should be included or excluded from the donor pool (although this also may be driven by theory or a researcher-developed conceptual model).

The objective aspect is:

- The systematic estimation of the weights used to construct the synthetic control from the units in the donor pool.

Clearly, the subjective aspects are important in this method. It is essential for the researcher to be happy that the control units within the donor pool are sufficiently similar to the treated unit. If this is the case, then a weighted combination of any of the units in the donor pool should be a credible counterfactual for the treated unit.

Box 2 **How synthetic control methodology (SCM) works**

1. The parameter of interest is the intervention effect for the treated unit, which is the difference between the outcome variable in the treated unit and the outcome variable in the synthetic control (SC) unit postintervention.
2. In order to construct a SC, data are required for the treated unit and for a number of similar units. The minimum data required is the outcome variable of interest for both treated and potential controls over at least one time point before and after the intervention, though in practice considerably more time points will be needed in order to improve the credibility of the result.
3. A counterfactual SC is constructed by weighting control units, such that the level and trend of the preintervention SC most closely matches the treated unit. SC aims to minimise the difference in the preintervention period in the predictors included between the intervention unit and a weighted average of control units. Predictors include preintervention values of the outcome as well as other important variables. The weights applied to different countries are based on those that minimise the difference (based on achieving the smallest root mean square prediction error). It is also usual to check that the preintervention trend in the outcome for the synthetic comparison closely matches that of the intervention. The usual default is to limit the individual country weights to being between 0 and 1 (with the total of the weights being 1) as weights outside this range would indicate extrapolation
4. Alternative approaches to the regression-based weighting of predictor variables are available, including researcher specified predictor variable weightings and simultaneous optimisation of predictor weights and control unit weights.
5. Once the weighting of the SC has been determined for the preintervention period, it is used to construct a counterfactual trend for the outcome in the postimplementation period. The weighting of the potential control units is the same over time. The difference between this counterfactual and the actual trend for the treated unit represents the estimated intervention effect.
6. Technical references are given in online supplementary material 4.

10.1136/jech-2017-210106.supp5 Supplementary file 5

### Advantages, assumptions and limitations of SCM

SCM has several advantages over alternative approaches to evaluating population health interventions. First, it offers an approach suitable when there is a small number of treated units and control units, which is often the case when population-level health interventions are being evaluated. Second, unlike DiD approaches, SCM does not rely on parallel preimplementation trends. Given that it is sometimes difficult to establish whether the parallel trends assumption is met, this method provides a useful supplementary method to DiD. Finally, SCM allows for unmeasured time-varying confounders, whereas DiD only allows for measured time-varying confounders. Any known time-varying confounder can be included in a DiD analysis as a time series variable. However, assuming the SC was constructed from a pool of similar units and a good fit was achieved over a sufficient period of time in the preimplementation period, the SCM accounts for both observed and unobserved time-varying confounding that might impact on the outcome of interest. Abadie *et al* assert that this is based on the ‘intuition’ that ‘only units that are alike in both observed and unobserved determinants of the outcome variable as well as in the effect of those determinants on the outcome variable should produce similar trajectories of the outcome variable over extended periods of time’.^3^

However, the credibility of the result relies on achieving a good preimplementation fit for the outcome of interest between treated unit and synthetic control, which is difficult if the treated unit is an outlier. It is also critical that the synthetic control is built from a pool of potential controls that are similar to the treated unit. There is currently no consensus on what constitutes a ‘good fit’ or how to judge similarity. Other assumptions necessary to the success of the method are that there are no ‘shocks’, that is, other events that might differentially affect the outcome of interest in the treated unit or the potential control units in either the preintervention or postintervention period and that there is no ‘contamination’ or spillover of the effect of the intervention into control units. These assumptions are also made by DiD approaches.

A limitation of SCM is that traditional statistical inference is inappropriate when there are small number of treated and control units (as is the case in many country and state level studies) and the fact that units are not sampled probabilistically. Alternative falsification tests have been suggested and two approaches are set out in the case study. Other approaches are being developed.^6 40^ Data availability in a consistent form across treated and control units may also prove to be a hurdle to the widespread adoption of the method.

Direct comparisons of SCM with other methods have produced mixed results. O’Neill *et al* 40 compared the performance of SCM against regression with lagged dependent variables and a hybrid matching/DiD approach in a study of pay for performance in UK healthcare. They concluded that SCM outperformed DiD when the parallel trends assumption was not met and that regression with lagged dependent variables outperformed SCM in most situations. They recommended the use of multiple methods. Online supplementary material 4 includes an example of a study that compared SCM with a propensity score-based weighting approach in the same context as Abadie *et al*’s study.^2^ The authors preferred the propensity score-based weighting method over SCM as it used familiar regression techniques, could be implemented using any basic statistical software and allowed greater flexibility in number of treated units and treatment effect estimators.

## Conclusion

SCM is a valuable addition to the range of approaches for improving causal inference in the evaluation of population level health interventions when a randomised trial is impractical. It has certain advantages over other more widely used approaches and is one of a small number of methods that may control for unmeasured time-varying confounders. Like all methods that draw causal inferences from observational data, SCM requires a number of assumptions and its applicability is limited by the requirement for series of outcome and covariate data on both the treated unit and a suitable pool of untreated comparison units. Wider use of SCM, ideally alongside other more established methods, will help to develop a better understanding of its strengths and limitations.

**What is already known on this subject**

- The synthetic control method can be used to evaluate population level health interventions.
- It uses a weighted combination of potential control units to act as a counterfactual (ie, what would have happened in the treated unit without the intervention).
- Potential control units must be similar to the treated unit and must not have been exposed to the intervention or have suffered other external shocks.
- It does not need an assumption of parallel trends like the difference in difference approach.

**What this study adds**

- A non-technical introduction to the method.
- A step-by-step guide to the implementation of the method with data and syntax provided.
- Examples of application to date of the method in health research.
- Reference to technical sources.

10.1136/jech-2017-210106.supp3 Supplementary file 3

10.1136/jech-2017-210106.supp4 Supplementary file 4
