# Supplementary material for: Synthetic control methodology as a tool for evaluating population-level health interventions
Source: J Epidemiol Community Health. 2018 Apr 13;72(8):673–8. doi: 10.1136/jech-2017-210106 (PMC6204967; doi:10.1136/jech-2017-210106)
Supplement: Supplementary file 1 [file jech-2017-210106supp001.pdf]

**Supplementary Materials 1 Details of literature search**

| | Result of search | Experimental' science studies excluded | Potentially relevant studies | Duplicates excluded | Abstracts screened | Reviewed for basic information | Reviewed in full | Of which related to health |
|---|---|---|---|---|---|---|---|---|
| **Database searched** | | | | | | | | |
| Proquest | 36 | 0 | 36 | 17 | 19 | 17 | 2 | 0 |
| Repec (Research Papers in Economics) | 111 | 0 | 111 | 68 | 43 | 35 | 8 | 7 |
| Econlit | 49 | 0 | 49 | 21 | 28 | 21 | 7 | 3 |
| Ovid Medline | 34 | 28 | 6 | 5 | 1 | 0 | 1 | 1 |
| Embase 1947-Present | 5 | 1 | 4 | 4 | 0 | 0 | 0 | 0 |
| Pubmed | 26 | 0 | 26 | 21 | 5 | 4 | 1 | 1 |
| ASSIA (American Psychological Association) | 6 | 0 | 6 | 4 | 2 | 0 | 2 | 2 |
| SocIndex | 9 | 0 | 9 | 9 | 0 | 0 | 0 | 0 |
| Sociological Abstracts | 3 | 0 | 3 | 1 | 2 | 1 | 1 | 1 |
| TRIP | 7 | 1 | 6 | 5 | 1 | 1 | 0 | 0 |
| CINAHL (Cumulative Index to Nursing and Allied Health) | 3 | 0 | 3 | 2 | 2 | 1 | 1 | 1 |
| Psychinfo | 11 | 4 | 7 | 5 | 2 | 1 | 1 | 1 |
| Business Source Premier | 21 | 0 | 21 | 5 | 16 | 15 | 1 | 1 |
| EBSCO Professional Development Collection | 3 | 0 | 3 | 3 | 0 | 0 | 0 | 0 |
| ERIC (Institute of Education Sciences) | 0 | 0 | 0 | 0 | 0 | 0 | 0 | 0 |
| National Institute for Health and Care Excellence (NICE) | 7 | 2 | 5 | 5 | 0 | 0 | 0 | 0 |
| **Other sources** | | | | | | | | |
| World Bank | 1 | 0 | 1 | 1 | 0 | 0 | 0 | 0 |
| RAND corporation | 1 | 0 | 1 | 0 | 1 | 0 | 1 | 0 |
| Citations of key articles | n/a | n/a | n/a | n/a | 17 | 11 | 6 | 4 |
| Author searches | n/a | n/a | n/a | n/a | 2 | 2 | 0 | 0 |
| References from studies reviewed | n/a | n/a | n/a | n/a | 7 | 3 | 7 | 5 |
| Google Scholar | 318 | 0 | 318 | 250 | 68 | 53 | 12 | 10 |
| What Works Scotland (note 1) | n/a | n/a | n/a | n/a | 8 | 6 | 2 | 1 |
| **Totals** | **651** | **36** | **615** | **426** | **224** | **171** | **53** | **38** |

Notes

1) Brief review of methodology published March 2015 - this review had access to some additional sources

2) For all searches except Google Scholar, Pubmed, Ovid Medline and Embase 1947-Present the search term used was "synthetic control$" in the full text". The search term was narrowed to "synthetic control$ method$" for Google Scholar, Pubmed, Ovid Medline and Embase 1947 as a large number of experimental science papers were returned with the wider term.
