# Supplementary material for: Synthetic control methodology as a tool for evaluating population-level health interventions
Source: J Epidemiol Community Health. 2018 Apr 13;72(8):673–8. doi: 10.1136/jech-2017-210106 (PMC6204967; doi:10.1136/jech-2017-210106)
Supplement: Supplementary file 2 [file jech-2017-210106supp002.pdf]

## Supplementary Materials 2

## Case study examining the impact of German reunification on life expectancy

Table A1 summarises our case study. This is a simplified analysis for illustration only and does not include the whole range of tests and comparisons with alternative methods that a robust analysis would require.

**Table A1: Outline of case-study** (OECD – Organisation of Economic Co-operation and Development. GDP – Gross Domestic Product)

| | |
|---|---|
| **Treated unit** | West Germany |
| **Potential control units** | OECD countries |
| **Outcome variable** | Life expectancy |
| **Predictor variable** | GDP |
| **Date of implementation** | 1990 |
| **Pre-implementation periods** | 1960-1989 |
| **Post-implementation periods** | 1990-2004 |

The syntax and data (Stata format) are available as Supplementary Materials. The syntax includes details on obtaining and constructing the dataset. The purpose of the case study is to use SCM to evaluate the impact of German reunification on life expectancy in West Germany by creating a synthetic control to represent life expectancy trends as if West Germany had not experienced reunification.

### Step 1 - Theoretical understanding

The first step in the SCM process is to make the theory and context around the intervention explicit. In particular, the characteristics chosen to match the real and synthetic treated units should be 'theory-driven' (i.e. factors associated with the outcome variable). Although the previous trend in the outcome variable can be the most important variable in determining the weighting of the potential control units in the synthetic control unit, it is important to include other predictor variables. This ensures that the synthetic control unit is as similar as possible in important characteristics as well as being able to replicate the pre-implementation trend for the outcome variable. In our simplified example we focus on GDP per capita as our additional predictor as this is hypothesised as being a possible cause of post reunification falls in life expectancy.

Detailed knowledge of the intervention is also necessary in order to ensure that any of the potential control units in the donor pool have not also been affected by the intervention. If a potential control unit has been affected by the intervention or similar interventions designed to achieve the same effect then the effect of the intervention may be diluted if the 'contaminated' unit is retained in the donor pool. Because reunification is such an unusual event we can be confident that the 'common shocks' assumption is satisfied.

### Step 2 – Identification of potential control units

The SCM builds a synthetic control from the values of the outcome and predictor variables of a range of potential controls known as 'the donor pool'. The initial donor pool tends to comprise a logical geographical or politico-economic grouping of countries or states such as all the states of the United States, countries of the OECD, or member states of the European Union. This is often because similar data are available for the grouping but also because membership of the group may suggest common characteristics. Our case study starts with the other 23 countries in the OECD at the time of German reunification. Potential control units that are not sufficiently similar to the treated unit should be excluded from the donor pool as the objective is to make a synthetic West Germany. If countries which are dissimilar to West Germany are retained in the pool there is more scope for unmeasured confounding. It may seem unimportant to exclude inappropriate potential controls from the pool as they would likely receive a zero weighting in the selection process. However, if the donor pool is large, the 'noise' from the outcome variable across the various countries may result in over-fitting in the pre-implementation period as virtually any trend could be matched. The resulting weights selected would not represent the best synthetic counterfactual. It should be noted that criteria for exclusion are set by the researcher and to date no clear consensus has been established as to what the process should be for excluding dissimilar units from the pool of potential donors. It is, therefore, essential to report clearly the criteria used and qualitatively assess the excluded units. Sensitivity analysis can be undertaken to determine how robust results are to various combinations of donor pool.

In their study of German reunification Abadie et al (reference 3 in the main manuscript) excluded 7 countries from the pool for reasons of size (Iceland and Luxembourg), much lower GDP (Turkey) and economic shocks in the post reunification period (Canada, Finland, Sweden, and Ireland). This left 16 countries in the pool Australia, Austria, Belgium, Denmark, France, Greece, Italy, Japan, the Netherlands, New Zealand, Norway, Portugal,

Spain, Switzerland, the United Kingdom, and the United States. We excluded Greece as our data source only had life expectancy for Greece back to 1981, leaving a pool of 15. We explored the trend in the mean life expectancy and GDP graphically (see Do file in Supplementary Materials) and the 5 country pool previously used was closer to the trend for West Germany. We, therefore, decided to restrict our pool to Austria, Japan, Switzerland, Netherlands and the United States. In a full analysis the researcher could conduct sensitivity analysis to determine the effect of this restriction.

### Step 3 – Develop the synthetic control country – a synthetic control West Germany.

Using data from the pre-reunification period only we use the Synthetic Control software to develop a weighted average from the 5 country pool. Reunification occurred in 1990 so the pre intervention period is 1960-1989. Importantly, we do not access outcome data post 1990 at this stage to reduce the likelihood of our synthetic control creation being influenced by the result. The aim is to minimise the difference in the outcome trend between West Germany and its synthetic control. This can be assessed visually, as well as being judged by calculating the difference between them. The synthetic control method minimises the root mean square prediction error (RMSPE) based on pre-intervention variables for those countries in the pool.

Given the aim to find the best fit to the outcome variable in the pre-intervention period and the fact that the researcher remains ‘blinded’ to the post-intervention outcome there is some flexibility in how to include the outcome variables in the analysis. Best practice on how to specify the variables is still lacking. Several approaches are possible including (1) using each year of the outcome variable as a separate variable; (2) using the final pre-intervention outcome variable; and (3),using an average over the pre-intervention period. The first approach is thought to lead to overfitting so will not be used here. We have used the second approach and a form of the third in this case study.

Using the values of the predictors (GDP and Life expectancy) at the end of the pre-intervention period (in our case 1989) gives the pre-intervention fit shown in the top row in Figure A1. Given the fit for GDP and life expectancy shown from the first approach we then looked at using some averages (i.e. the third approach). As in our first iteration above, the match for GDP per capita was good until roughly 1967 and for life expectancy until 1983 we included variables representing each of these periods. Overall for life expectancy we included three variables (average from 1960 to 1969, from 1970 to 1982 and from 1983 to 1989) and for GDP per capita we included two (1960 to 1966 and 1967 to 1989). Hence there are now

five variables. The bottom row of Figure A1 shows the fit of this specification and the RMSPE and weightings of predictor variables and control units are shown in Table A2.

**Figure A1: Two approaches to finding a synthetic control for West Germany's life expectancy in the pre-implementation period (pre 1990).** The top row shows the results of fitting using only the 1989 values for life expectancy and Gross Domestic Product (GDP). The second row shows the results when averages are used for life expectancy (from 1960 to 1969, from 1970 to 1982 and from 1983 to 1989) and for GDP per capita (1960 to 1966 and 1967 to 1989).

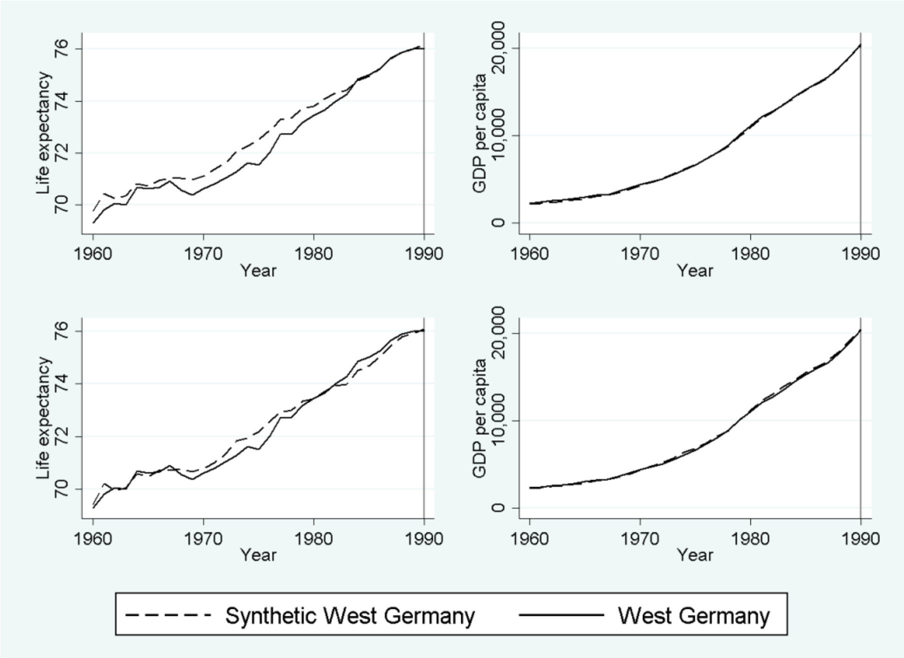


The two approaches give RMSPE, weightings of predictor variables and country weightings as set out in Table A2.

**Table A2: Root Mean Square Prediction Error (RMSPE), weightings of predictor variables and country weightings** (GDP: Gross Domestic Product)

| Approach | Using 1989 GDP and life expectancy | Using averages |
|---|---|---|
| RMSPE | 0.46 | 0.26 |
| *Weighting of variables* | | |
| Life expectancy | 95% | 32% |
| GDP | 5% | 68% |
| | | |
| *Weighting of control units* | | |
| Austria | 51% | 70% |
| Japan | 6% | - |
| Netherlands | 14% | - |
| Switzerland | 9% | 19% |
| USA | 20% | 11% |

The averaging approach produces a better pre-implementation fit so this was the approach we adopted. Although it may feel inappropriate to be 'exploring' different approaches to finding pre-implementation fit, recall that we are still blinded to the post-implementation data and that each of the countries in the control group is a close match to West Germany so may have been considered an appropriate control in a case comparison type study. In a full analysis we would include more predictors and try and further minimise the RMPSE.

### Step 4 – Run outcome analysis

Once an acceptable fit has been obtained between actual and synthetic West Germany, the analysis is run using the full pre and post-implementation data set, presented graphically and results retained for robustness checks.

### Step 5 - Present results

A figure (Figure A2) is produced showing the real and synthetic West Germany graphically with a line at the date of the intervention. Ideally, there should be a good fit to the left of the line in the pre-intervention period (from stages 1 to 3) and, if the intervention has an effect, there should be a gap between the real and synthetic unit after the date of the intervention. In the case of life expectancy there appears to be a small lower life expectancy post reunification in West Germany than its synthetic counterpart. The gap at particular points in time or over the full post-implementation period can be calculated arithmetically. The reduction in life expectancy at the end of the post-implementation period (2003) in West

Germany was 0.4 years, so any effect, if real, is small. The figure also confirms the previous result that GDP fell after reunification.

**Figure A2 Life expectancy and GDP for West Germany and its synthetic control** (GDP: Gross Domestic Product, vertical line represents the date of the implementation)

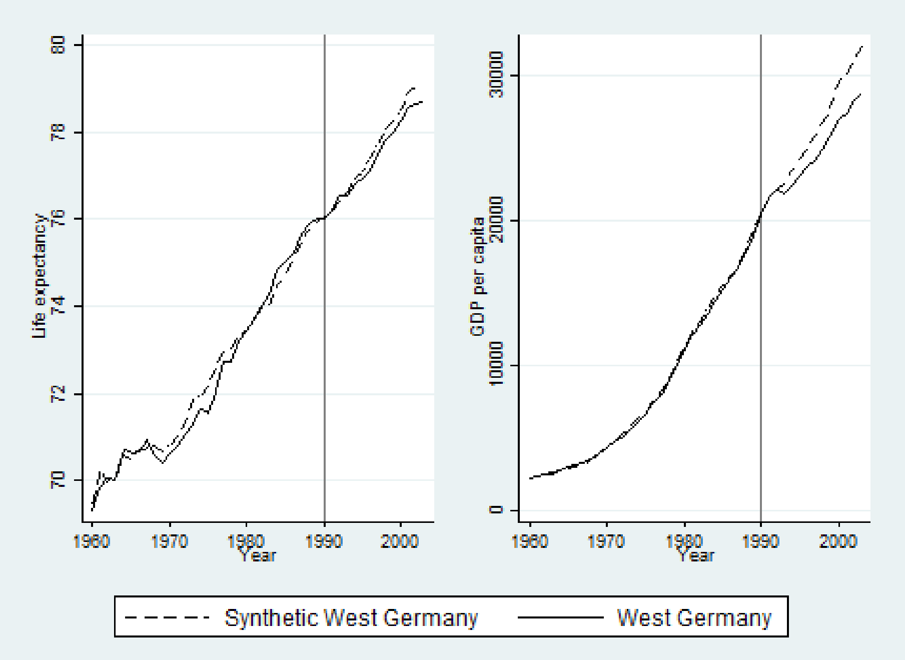


### Step 6 – Run robustness checks

What confidence should we place in the life expectancy results? Standard statistical inference is not appropriate with SCM as there are generally insufficient units to estimate a distribution, there is no randomization between treated and control, and probabilistic sampling is not employed to select sample units. Instead various robustness checks can be undertaken to improve causal inference.  The most common check is known as an 'in-space placebo' test where each potential control unit in turn is used as the treated unit and its own synthetic control developed (from a pool excluding the actual treated country).  If the gap for the 'real' treated unit is a true intervention effect then the gap should be large compared to the gap for all the placebo analyses. This is not the case in our example.

**Figure A3: In-space placebo analysis.** The dark line represents the gap between the life-expectancy for West Germany and its synthetic control, the lighter lines represent the gaps when the analysis is run using each other country in the donor pool as the treated unit. The vertical line represents the date of implementation.

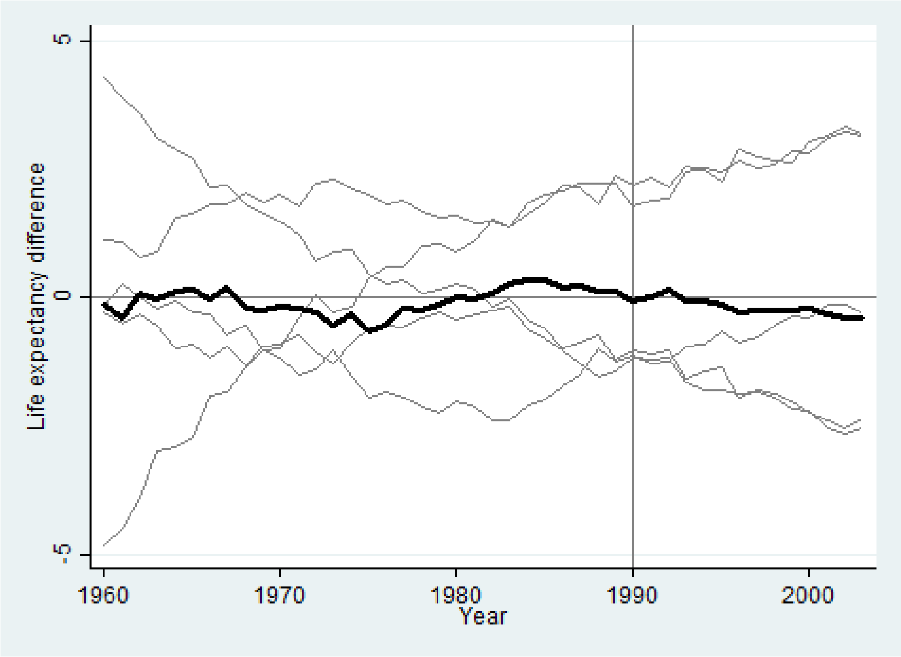


It is also possible to do a similar falsification exercise using other times of implementation or other outcomes (in-time and in-outcome placebo tests). Another sensitivity-type test undertaken in some studies using SCM includes ‘sparse synthetic control’, where the number of control units is reduced from the original specification step by step and the effect on weighting of donor units and quality of pre-implementation fit assessed. Although only one of the health-related studies used this approach it is useful to gauge the difference in result using the weighted synthetic control compared to a single control unit. A further possibility is ‘leave one out’ analysis where each country making up the control unit is removed one at a time from the donor pool. This allows the sensitivity of the result to the inclusion of a particular control unit to be assessed.

A further stage in the placebo analysis is to calculate a ratio of the post-implementation RMSPE to the pre-intervention RMSPE. This is a measure of how big the gap between real treated unit and synthetic control is after the intervention date as a function of the gap before the intervention. If the fit between the actual and synthetic treated units before the intervention is not good the ratio will be smaller. The larger the ratio, the more convincing the evidence that the intervention has had an effect. As the table below shows West Germany’s RMSPE ratio is not the lowest. However confirming the visual results in Figure

A3 this is not because of a poor pre intervention fit but because post intervention it is little changed.

**Table A3: Pre and post implementation Root Mean Square Prediction Error (RMSPE) for West Germany and other countries in the donor pool (in-space placebo analysis)**

| | Pre implementation RMSPE | Post implementation RMSPE | Ratio post / pre |
|---|---|---|---|
| | | | |
| **Austria** | 1.51 | 0.82 | 0.55 |
| **Japan** | 2.08 | 2.81 | 1.36 |
| **Netherlands** | 1.80 | 1.91 | 1.06 |
| **Switzerland** | 1.79 | 2.70 | 1.51 |
| **USA** | 0.86 | 2.03 | 2.36 |
| **West Germany** | 0.26 | 0.25 | 0.96 |
