# Supplementary material for: Synthetic control methodology as a tool for evaluating population-level health interventions
Source: J Epidemiol Community Health. 2018 Apr 13;72(8):673–8. doi: 10.1136/jech-2017-210106 (PMC6204967; doi:10.1136/jech-2017-210106)
Supplement: Supplementary file 5 [file jech-2017-210106supp005.pdf]

SUPPLEMENTARY MATERIALS 4: TECHNICAL PAPERS

References relating to evaluation of public health interventions

[1] Hawe P, Potvin L. What is population health intervention research? Can J Public Health. 2009 Jan-Feb;100(1):Suppl I8-14.

[2] Bor J. Capitalizing On Natural Experiments To Improve Our Understanding Of Population Health. Am J Public Health 106.8 (2016): 1388-1389.

[3] Craig P, Dieppe P, Macintyre S, Michie S, Nazareth I and Petticrew M. Developing and evaluating Complex Interventions: The New Medical Research Council Guidance. BMJ (2008): a1655.

[4] Jones AM and Rice N. Econometric evaluation of health policies. In Glied S, Smith P (eds.), The Oxford Handbook of Health Economics, Oxford University Press: Oxford, UK. 2011.

[5] Lawlor DA, Tilling K and Davey Smith. Triangulation in aetiological epidemiology. Int J Epidemiol,(2017), 1-21 doi: 10.1093/ije/dyw314

[6] Craig P, Katikireddi SV, Leyland A and Popham F. Natural Experiments: An Overview of Methods, Approaches, and Contributions to Public Health Intervention Research. Annual Review of Public Health 2017 38:1, 39-56

References to papers offering critique of the synthetic control method

[1] Dube A and Zipperer B, Pooled Synthetic Control Estimates for Recurring Treatment: An Application to Minimum Wage Studies, University of Massachusetts, Amherst working paper, 2013

This paper explores issues relating to the extension of the method into multiple treated units.

[2] Kloβner S, Kaul A, Pfeifer G and Shieler M, 2015, Comparative Politics and the Synthetic Control Method Revisited: A note on Abadie et al. Working paper. Saarland University Available from http://www.oekonometrie.uni-saarland.de/papers/Cross-Validation.pdf

This study re-examines the 2014 Abadie et al study [reference 3 in the main manuscript] and points out some limitations in the methodology and current software.

[3] Fremeth A, Holburn G, Richter B (2016) Bridging Qualitative and Quantitative Methods in Organizational Research: Applications of Synthetic Control Methodology in the U.S. Automobile Industry. Organization Science 27(2):462-482. Available at http://dx.doi.org/10.1287/orsc.2015.1034

This paper sets out a good overview of the method and comparison with other methods of estimating treatment effect.

[4] Linden A and Adams JL. Applying A Propensity Score-Based Weighting Model To Interrupted Time Series Data: Improving Causal Inference In Programme Evaluation. J Eval Clin Pract 17.6 (2010): 1231-1238.

This study compared SCM with a propensity score-based weighting approach in the same context as Abadie et al’s 2010 study. [reference 2 in the main manuscript]
