# Supplementary material for: Synthetic control methodology as a tool for evaluating population-level health interventions
Source: J Epidemiol Community Health. 2018 Apr 13;72(8):673–8. doi: 10.1136/jech-2017-210106 (PMC6204967; doi:10.1136/jech-2017-210106)
Supplement: Supplementary file 3 [file jech-2017-210106supp003.pdf]

```
1  * Frank Popham & Janet Bouttell - 27/06/2017 licensed as https://creativecommons.org/licenses/by/4.0/
2
3  **Lines 9-67 cover the creation of the dataset**
4
5  **If preferred you can go directly to line 70**
6  **You will, however, need to install synth (see line 19), synth_runner (see line 22) and grc1leg(see line 33)**
7
8
9  *Data sources: Life expectancy: Human Mortality Database.  University of California, Berkeley (USA), and Max Planck Institute for Demographic Research (Germany). Available at www.mortality.org or www.humanmortality.de (data downloaded on 29/03/2017).
10 *all other:  Hainmueller, Jens, 2014, "Replication data for: Comparative Politics and the Synthetic Control Method", doi:10.7910/DVN/24714, Harvard Dataverse, V2, UNF:5:AtEF45hDnFLetMIiv9tjpQ==  (data downloaded on 29/03/2017).
11
12 *Combining datasets
13 *First import txt files from HMD into stata, keep years 1960 to 2003, The countries included (as per Comparative Politics and the Synthetic Control Method are  Australia
14 *Austria, Belgium, Denmark, France, Greece, Italy, Japan, Netherlands,New Zealand, Norway, Portugal, Spain, Switzerland, UK, USA, West Germany. Greece only starts 1981 in HMD so we exclude)
15
16 *If not already installed you will need to install the following
17
18 *synth - https://web.stanford.edu/~jhain/synthpage.html
19 ssc install synth, replace all
20
21 *synth_runner Brian Quistorff and Sebastian Galiani. The synth_runner package: Utilities to automate synthetic control estimation using synth, Mar 2017. https://github.com/bquistorff/synth_runner. Version 1.4.0.
22 net install synth_runner, from( "https://raw.github.com/bquistorff/synth_runner/master/") replace
23
24 *dsconcat - Roger Newson, Imperial College London, UK.
25 ssc install dsconcat, replace
26
27 *kountry -  Raciborski, R. (2008). "kountry: A Stata utility for merging cross-country data from multiple sources," The Stata Journal, 8(3), 390-400.
28
29 ssc install kountry, replace
30
31 *grc1leg Program by Vince Wiggins, StataCorp <vwiggins@stata.com>.
32
33 net install grc1leg.pkg, replace
34
35 *assumes hmd files (for  year on year life expectancy for above countries) in a directory on their own
36 cd E0per
37 *imports text files and saves them as stata files
38 local list : dir "." files "*.txt", respectcase
39 foreach file of local list {
40 import delimited `file', varnames(3)  delimiters(" ", collapse) clear
41 gen filename="`file'"
42 gen country=substr(filename,1,3)
43 levelsof country
44 keep year country total male female
45 drop if year < 1960
46 drop if year > 2003
47 save `r(levels)', replace
48  }
49 *combines the imported stata  files from the last step (uses user written programme dsconcat so if you don't have this use the * out command to install from ssc).
50   local list2 : dir "." files "*.dta", respectcase
51
52 dsconcat `list2'
53
54 *next merge the files using a user written programme - kountry- again download this if haven't already
55
56 kountry country, from(iso3c)
```

```
57   save "germany\le", replace
58   cd "germany"
59   use repgermany, clear
60   kountry country, from(other)
61   drop if country=="Greece"
62   merge 1:1 NAMES_STD year using le
63   drop _merge male female
64   encode country, gen(country2)
65   label var total "Life expectancy"
66   save analysis, replace
67
68   *****************************
69   *
70   * Analysis - start here
71   *
72   *****************************
73   use analysis, clear
74   *Step 1  - requires no syntax as it concerns theoretical understanding*
75   *Step 2 - Identification of potential control units - remaining blinded to data
     post implementation*
76   keep if year < 1991
77
78   *exclusions - keep Austria, Japan, Netherlands, Switzerland and USA as well as West
     Germany as these used in GDP study.
79
80   *compares West German trend to the mean of the rest of the 15
81   egen m_total = mean(total) if country2!=16, by(year)
82
83   egen m_gdp = mean(gdp) if country2!=16, by(year)
84
85   *pool after exclusions
86   egen m_total_ex = mean(total) if inlist(country2,2,7,8,13,15) ,  by(year)
87
88   egen m_gdp_ex = mean(gdp) if inlist(country2,2,7,8,13,15) , by(year)
89
90
91   *Comparison of average GDP and Life Expectancy trends between West Germany and 5
     country/15 country pools*
92   line m_total year if country2==2, lpattern(dash) lcolor(black) || line  m_total_ex
     year if country2==2, lpattern(dash_dot) lcolor(black) ||line total year if country2
     ==16, name(match_le, replace)  ytitle("Life expectancy") xline(1990, lcolor(gs8)) ///
93   legend(label(1 "15 country pool") label(2 "5 country pool") label(3 "West Germany"))
      lcolor(black)
94
95   line m_gdp year if country2==2, lpattern(dash) lcolor(black) || line  m_gdp_ex year
     if country2==2, lpattern(dash_dot) lcolor(black)  || line gdp year if country2==16 &
      year, name(match_gdp, replace)  ytitle("GDP per capita") xline(1990, lcolor(gs8))
     ///
96   legend(label(1 "15 country pool") label(2 "5 country pool") label(3 "West Germany"))
      lcolor(black)
97
98   grc1leg match_le match_gdp, xcommon
99
100  *Figure generated by line 98 (not shown in article) shows better GDP fit for 5
     country pool so that is used in the rest of the analysis*
101  keep if inlist(country2,2,7,8,13,15,16)
102
103  *Step 3 - Develop the synthetic control country - a synthetic control West Germany
104
105  tsset country2 year
106
107
108  **This approach uses the final gdp observation in the pre-implementation period as
     predictor variable**
109  **Lines 112-124 create the top half of Figure 1**
110  synth total total(1989) gdp(1989), trunit(16) trperiod(1990)
111
112  sort year country2
113  matrix W = e(W_weights)
114  svmat W
115  bysort country2: egen weight = max(W2)
116  egen m_gdp_temp = total(gdp*weight) if country2!=16 & weight > 0, by(year)
117  egen m_total_temp = total(total*weight) if country2!=16 & weight > 0, by(year)
```

```
118 sort country2 year
119 line m_gdp_temp year if country2==2, lpattern(dash) lcolor(black) || line gdp year
    if country2==16, ///
120 legend(label(1 "Synthetic West Germany") label(2 "West Germany"))  name(match_gdp_1,
     replace)  ytitle("GDP per capita") xline(1990, lcolor(gs8)) lcolor(black) ylabel(0
    "0" 10000"10,000" 20000"20,000")
121 line m_total_temp year if country2==2, lpattern(dash) lcolor(black) || line total
    year if country2==16, ///
122 legend(label(1 "Synthetic West Germany") label(2 "West Germany"))  name(match_le_1,
    replace)  ytitle("Life expectancy") xline(1990, lcolor(gs8)) lcolor(black)
123 drop W* weight m_gdp_temp m_total_temp
124 matrix list e(V_matrix)
125
126 **This approach uses averages of GDP over five-year periods in the
    pre-implementation period as predictor variables**
127 **Lines 128-142 create the bottom half of Figure 1**
128 synth total total(1983(1)1989) total(1970(1)1982) total(1960(1)1969) gdp(1960(1)1966
    ) gdp(1967(1)1989), trunit(16) trperiod(1990)
129
130 sort year country2
131 matrix W = e(W_weights)
132 svmat W
133 bysort country2: egen weight = max(W2)
134 egen m_gdp_temp = total(gdp*weight) if country2!=16 & weight > 0, by(year)
135 egen m_total_temp = total(total*weight) if country2!=16 & weight > 0, by(year)
136 sort country2 year
137 line m_gdp_temp year if country2==2, lpattern(dash) lcolor(black) || line gdp year
    if country2==16, ///
138 legend(label(1 "Synthetic West Germany") label(2 "West Germany"))  name(match_gdp_2,
     replace)  ytitle("GDP per capita") xline(1990, lcolor(gs8)) lcolor(black) ylabel(0
    "0" 10000"10,000" 20000"20,000")
139 line m_total_temp year if country2==2, lpattern(dash) lcolor(black) || line total
    year if country2==16, ///
140 legend(label(1 "Synthetic West Germany") label(2 "West Germany"))  name(match_le_2,
    replace)  ytitle("Life expectancy") xline(1990, lcolor(gs8)) lcolor(black)
141 matrix list e(V_matrix)
142 drop W* weight m_gdp_temp m_total_temp
143
144 **Line 145 creates Figure 1**
145 grc1leg match_le_1 match_gdp_1 match_le_2 match_gdp_2, xcommon
146
147 **Step 4 - Run outcome analysis**
148
149 use analysis, clear
150 set more off
151
152 keep if inlist(country2,2,7,8,13,15,16)
153
154
155 **Lines 156-172 create Figure 2**
156 tsset country2 year
157 synth total total(1983(1)1989) total(1970(1)1982) total(1960(1)1969) gdp(1960(1)1966
    ) gdp(1967(1)1989) ///
158 , trunit(16) trperiod(1990)
159 sort year country2
160 matrix W = e(W_weights)
161 svmat W
162 bysort country2: egen weight = max(W2)
163 egen m_gdp_temp = total(gdp*weight) if country2!=16 & weight > 0, by(year)
164 egen m_total_temp = total(total*weight) if country2!=16 & weight > 0, by(year)
165 sort country2 year
166 line m_gdp_temp year if country2==2, lpattern(dash) lcolor(black)  || line gdp year
    if country2==16, ///
167 legend(label(1 "Synthetic West Germany") label(2 "West Germany"))  name(match_gdp,
    replace)  ytitle("GDP per capita") xline(1990, lcolor(gs8)) lcolor(black)
168 line m_total_temp year if country2==2, lpattern(dash) lcolor(black) || line total
    year if country2==16, ///
169 legend(label(1 "Synthetic West Germany") label(2 "West Germany"))  name(match_le,
    replace)  ytitle("Life expectancy") xline(1990, lcolor(gs8)) lcolor(black)
170 grc1leg match_le match_gdp, xcommon
171 drop W* weight m_gdp_temp m_total_temp
172 matrix list e(V_matrix)
173
```

```
174 **Step 6 - Run robustness checks**
175 **Lines 176-186 create Figure 3**
176 tempfile keepfile
177 synth_runner total total(1983(1)1989) total(1970(1)1982) total(1960(1)1969) gdp(1960(1)1966) gdp(1967(1)1989) ///
178 , trunit(16) trperiod(1990) keep(`keepfile')
179 merge 1:1 country2 year using "`keepfile'", nogenerate
180 gen double total_synth = total-effect
181 line effect year if country2 == 2, lcolor(gs8) || ///
182 line effect year if country2 == 7, lcolor(gs8) || ///
183 line effect year if country2 == 8, lcolor(gs8) || ///
184 line effect year if country2 == 13, lcolor(gs8) || ///
185 line effect year if country2 == 15, lcolor(gs8) || ///
186 line effect year if country2 == 16, lcolor(gs0) legend(off)  lwidth(thick) xline(1990, lcolor(gs8)) yline(0, lcolor(gs8)) ytitle(Life expectancy difference)
187
188 *RMPSE
189 **Lines 190-191 generate the data for Table 4**
190 gen ratio_rmspe = post_rmspe / pre_rmspe
191 tabstat pre_rmspe post_rmspe ratio_rmspe, by(country2) nototal
192
193 **Further sensitivity analysis - not discussed in article**
194
195 *Do exclusions matter?
196
197 use analysis, clear
198 set more off
199
200 tsset country2 year
201 synth total total(1983(1)1989) total(1970(1)1982) total(1960(1)1969) gdp(1960(1)1966) gdp(1967(1)1989) , trunit(16) trperiod(1990)
202 sort year country2
203 matrix W = e(W_weights)
204 svmat W
205 bysort country2: egen weight = max(W2)
206 egen m_gdp_temp = total(gdp*weight) if country2!=16 & weight > 0, by(year)
207 egen m_total_temp = total(total*weight) if country2!=16 & weight > 0, by(year)
208 sort country2 year
209 line m_gdp_temp year if country2==2, lpattern(dash) lcolor(black) || line gdp year if country2==16, ///
210 legend(label(1 "Synthetic West Germany") label(2 "West Germany"))  name(match_gdp, replace)  ytitle("GDP per capita") xline(1990, lcolor(gs8)) lcolor(black)
211 line m_total_temp year if country2==2, lpattern(dash) lcolor(black) || line total year if country2==16, ///
212 legend(label(1 "Synthetic West Germany") label(2 "West Germany"))  name(match_le, replace)  ytitle("Life expectancy") xline(1990, lcolor(gs8)) lcolor(black)
213 grc1leg match_le match_gdp, xcommon
214 matrix list e(V_matrix)
215
216 tempfile keepfile
217 synth_runner total total(1983(1)1989) total(1970(1)1982) total(1960(1)1969) gdp(1960(1)1966) gdp(1967(1)1989) ///
218 , trunit(16) trperiod(1990) keep(`keepfile')
219 merge 1:1 country2 year using "`keepfile'", nogenerate
220 gen double total_synth = total-effect
221 line effect year if country2 == 1, lcolor(gs8) || ///
222 line effect year if country2 == 2, lcolor(gs8) || ///
223 line effect year if country2 == 3, lcolor(gs8) || ///
224 line effect year if country2 == 4, lcolor(gs8) || ///
225 line effect year if country2 == 5, lcolor(gs8) || ///
226 line effect year if country2 == 6, lcolor(gs8) || ///
227 line effect year if country2 == 7, lcolor(gs8) || ///
228 line effect year if country2 == 8, lcolor(gs8) || ///
229 line effect year if country2 == 9, lcolor(gs8) || ///
230 line effect year if country2 == 10, lcolor(gs8) || ///
231 line effect year if country2 == 11, lcolor(gs8) || ///
232 line effect year if country2 == 12, lcolor(gs8) || ///
233 line effect year if country2 == 13, lcolor(gs8) || ///
234 line effect year if country2 == 14, lcolor(gs8) || ///
235 line effect year if country2 == 15, lcolor(gs8) || ///
236 line effect year if country2 == 16, lcolor(gs0) legend(off)  lwidth(thick) xline(1990) yline(0) ytitle(Effect size)
237
```

```
*RMPSE
gen ratio_rmspe = post_rmspe / pre_rmspe
tabstat pre_rmspe post_rmspe ratio_rmspe, by(country2) nototal
```
