# Supplementary material for: Synthetic control methodology as a tool for evaluating population-level health interventions
Source: J Epidemiol Community Health. 2018 Apr 13;72(8):673–8. doi: 10.1136/jech-2017-210106 (PMC6204967; doi:10.1136/jech-2017-210106)
Supplement: Supplementary file 4 [file jech-2017-210106supp004.pdf]

| | | | | | | | | | | | | | |
|---|---|---|---|---|---|---|---|---|---|---|---|---|---|
| 2 | UK | 1960 | 2158 | | 43.25547 | 24.5 | | | | | United King | 71.04 | UK |
| 2 | UK | 1961 | 2216 | 3.579418 | 41.53324 | | | | | | United King | 70.79 | UK |
| 2 | UK | 1962 | 2276 | 4.103672 | 40.40463 | | | | | | United King | 70.85 | UK |
| 2 | UK | 1963 | 2386 | 2.074689 | 40.40659 | | | | | | United King | 70.76 | UK |
| 2 | UK | 1964 | 2534 | 3.211382 | 40.51876 | | | | | | United King | 71.55 | UK |
| 2 | UK | 1965 | 2660 | 4.805041 | 39.22534 | 26.5 | | | | | United King | 71.52 | UK |
| 2 | UK | 1966 | 2794 | 3.908305 | 38.96463 | | | | | | United King | 71.44 | UK |
| 2 | UK | 1967 | 2923 | 2.423146 | 39.31319 | | | | | | United King | 72.07 | UK |
| 2 | UK | 1968 | 3173 | 4.731638 | 43.66513 | | | | | | United King | 71.69 | UK |
| 2 | UK | 1969 | 3391 | 5.461902 | 44.03898 | | | | | | United King | 71.66 | UK |
| 2 | UK | 1970 | 3615 | 6.361893 | 45.24774 | 28.9 | | | | | United King | 71.9 | UK |
| 2 | UK | 1971 | 3854 | 9.407875 | 44.9334 | | | | | 43.07859 | United King | 72.23 | UK |
| 2 | UK | 1972 | 4149 | 7.087912 | 43.55904 | | | | | 42.56008 | United King | 72 | UK |
| 2 | UK | 1973 | 4683 | 9.209851 | 49.80358 | | | | | 42.1354 | United King | 72.19 | UK |
| 2 | UK | 1974 | 5036 | 15.90322 | 60.96212 | | | | | 41.62375 | United King | 72.38 | UK |
| 2 | UK | 1975 | 5481 | 24.23508 | 53.56206 | 31 | | | | 40.48818 | United King | 72.65 | UK |
| 2 | UK | 1976 | 5953 | 16.53255 | 58.16979 | | | | | 41.08992 | United King | 72.64 | UK |
| 2 | UK | 1977 | 6489 | 15.88198 | 59.42042 | | | | | 41.33041 | United King | 73.12 | UK |
| 2 | UK | 1978 | 7176 | 8.215962 | 55.65679 | | | | | 41.46581 | United King | 73.06 | UK |
| 2 | UK | 1979 | 7973 | 13.46571 | 55.69035 | | | | | 41.82387 | United King | 73.16 | UK |
| 2 | UK | 1980 | 8502 | 17.97059 | 52.32687 | 32.9 | 0.2008 | 0.22602 | 17.588 | 42.3463 | United King | 73.59 | UK |
| 2 | UK | 1981 | 9161 | 11.87983 | 50.51421 | | | | | 41.89466 | United King | 73.92 | UK |
| 2 | UK | 1982 | 9917 | 8.590529 | 50.75241 | | | | | 41.49169 | United King | 74.04 | UK |
| 2 | UK | 1983 | 10669 | 4.607018 | 52.0913 | | | | | 40.90145 | United King | 74.29 | UK |
| 2 | UK | 1984 | 11336 | 4.953409 | 57.00433 | | | | | 40.38236 | United King | 74.68 | UK |
| 2 | UK | 1985 | 12068 | 6.065421 | 56.62236 | 35.7 | | | | 39.76278 | United King | 74.53 | UK |
| 2 | UK | 1986 | 12795 | 3.427615 | 52.0564 | | | | | 39.13434 | United King | 74.8 | UK |
| 2 | UK | 1987 | 13717 | 4.148918 | 51.93616 | | | | | 38.03052 | United King | 75.15 | UK |
| 2 | UK | 1988 | 14864 | 4.907976 | 49.64918 | | | | | 37.21299 | United King | 75.26 | UK |
| 2 | UK | 1989 | 15716 | 7.797271 | 51.64563 | | | | | 36.62967 | United King | 75.4 | UK |
| 2 | UK | 1990 | 16397 | 9.475588 | 51.46746 | 38.5 | | | | 35.17424 | United King | 75.74 | UK |
| 2 | UK | 1991 | 16681 | 5.853981 | 48.20938 | | | | | 33.12701 | United King | 75.91 | UK |
| 2 | UK | 1992 | 17069 | 3.732601 | 49.34976 | | | | | 31.88967 | United King | 76.3 | UK |
| 2 | UK | 1993 | 17845 | 1.564474 | 52.59363 | | | | | 31.27093 | United King | 76.16 | UK |
| 2 | UK | 1994 | 18975 | 2.47645 | 54.25776 | | | | | 31.69333 | United King | 76.72 | UK |
| 2 | UK | 1995 | 19860 | 3.410996 | 57.89684 | 39.9 | | | | 31.99648 | United King | 76.62 | UK |
| 2 | UK | 1996 | 20923 | 2.448706 | 59.84958 | | | | | 31.86726 | United King | 76.88 | UK |
| 2 | UK | 1997 | 22280 | 3.132333 | 57.84053 | | | | | 31.07413 | United King | 77.14 | UK |
| 2 | UK | 1998 | 23206 | 3.41817 | 55.98906 | | | | | 29.46092 | United King | 77.28 | UK |
| 2 | UK | 1999 | 23959 | 1.555385 | | | | | | 28.59877 | United King | 77.38 | UK |
| 2 | UK | 2000 | 25583 | 2.9271 | | 39.1 | | | | 28.47661 | United King | 77.86 | UK |
| 2 | UK | 2001 | 27026 | 1.820852 | | | | | | 27.47552 | United King | 78.13 | UK |
| 2 | UK | 2002 | 28969 | 1.634458 | | | | | | 26.99038 | United King | 78.25 | UK |
| 2 | UK | 2003 | 29609 | 2.913632 | | | | | | 26.59016 | United King | 78.34 | UK |
| 1 | USA | 1960 | 2879 | | 9.693181 | 43.8 | | | | | United Stat | 69.83 | USA |
| 1 | USA | 1961 | 2929 | 1.075182 | 9.444654 | | | | | | United Stat | 70.24 | USA |
| 1 | USA | 1962 | 3103 | 1.116071 | 9.429324 | | | | | | United Stat | 70.11 | USA |
| 1 | USA | 1963 | 3227 | 1.214128 | 9.470706 | | | | | | United Stat | 69.94 | USA |
| 1 | USA | 1964 | 3420 | 1.308615 | 9.725879 | | | | | | United Stat | 70.19 | USA |
| 1 | USA | 1965 | 3667 | 1.668461 | 9.730347 | 43.8 | | | | | United Stat | 70.24 | USA |
| 1 | USA | 1966 | 3974 | 2.991 | 10.09759 | | | | | | United Stat | 70.21 | USA |
| 1 | USA | 1967 | 4154 | 2.775636 | 10.2081 | | | | | | United Stat | 70.52 | USA |
| 1 | USA | 1968 | 4494 | 4.217721 | 10.59394 | | | | | | United Stat | 70.22 | USA |
| 1 | USA | 1969 | 4805 | 5.414701 | 10.63563 | | | | | | United Stat | 70.48 | USA |
| 1 | USA | 1970 | 4999 | 5.895296 | 11.38864 | 44.4 | | | | | United Stat | 70.74 | USA |
| 1 | USA | 1971 | 5362 | 4.255929 | 11.36758 | | | | | 34.50863 | United Stat | 71.09 | USA |
| 1 | USA | 1972 | 5838 | 3.305615 | 11.96716 | | | | | 34.44429 | United Stat | 71.18 | USA |
| 1 | USA | 1973 | 6464 | 6.220064 | 13.71356 | | | | | 34.37521 | United Stat | 71.4 | USA |
| 1 | USA | 1974 | 6951 | 11.03526 | 17.31614 | | | | | 34.03606 | United Stat | 71.97 | USA |
| 1 | USA | 1975 | 7519 | 9.13193 | 16.37969 | 42.6 | | | | 33.22567 | United Stat | 72.54 | USA |
| 1 | USA | 1976 | 8300 | 5.737027 | 16.87366 | | | | | 33.76085 | United Stat | 72.85 | USA |
| 1 | USA | 1977 | 9146 | 6.48646 | 17.22059 | | | | | 34.26464 | United Stat | 73.22 | USA |
| 1 | USA | 1978 | 10229 | 7.647464 | 17.80619 | | | | | 34.03723 | United Stat | 73.42 | USA |
| 1 | USA | 1979 | 11306 | 11.26604 | 19.28055 | | | | | 33.62648 | United Stat | 73.83 | USA |
| 1 | USA | 1980 | 12186 | 13.50937 | 21.08158 | 62.9 | 0.23884 | 0.24568 | 22.376 | 33.51439 | United Stat | 73.74 | USA |
| 1 | USA | 1981 | 13533 | 10.31553 | 20.36324 | | | | | 34.08828 | United Stat | 74.13 | USA |
| 1 | USA | 1982 | 13940 | 6.160616 | 18.47162 | | | | | 32.90969 | United Stat | 74.47 | USA |
| 1 | USA | 1983 | 15008 | 3.212435 | 17.62431 | | | | | 31.49722 | United Stat | 74.56 | USA |
| 1 | USA | 1984 | 16549 | 4.317269 | 18.57701 | | | | | 31.87278 | United Stat | 74.69 | USA |
| 1 | USA | 1985 | 17600 | 3.561116 | 17.64241 | 57.4 | | | | 30.86452 | United Stat | 74.67 | USA |
| 1 | USA | 1986 | 18439 | 1.858736 | 17.95412 | | | | | 29.44326 | United Stat | 74.75 | USA |
| 1 | USA | 1987 | 19407 | 3.740876 | 19.11619 | | | | | 29.19973 | United Stat | 74.88 | USA |
| 1 | USA | 1988 | 20711 | 4.009088 | 20.33082 | | | | | 29.60649 | United Stat | 74.86 | USA |
| 1 | USA | 1989 | 22047 | 4.827003 | 20.73029 | | | | | 28.61904 | United Stat | 75.13 | USA |
| 1 | USA | 1990 | 23064 | 5.397956 | 21.18795 | 44.4 | | | | 27.85032 | United Stat | 75.4 | USA |
| 1 | USA | 1991 | 23507 | 4.234964 | 21.26635 | | | | | 26.62006 | United Stat | 75.55 | USA |
| 1 | USA | 1992 | 24509 | 3.02882 | 21.54221 | | | | | 25.72843 | United Stat | 75.81 | USA |
| 1 | USA | 1993 | 25409 | 2.951657 | 21.55765 | | | | | 25.61001 | United Stat | 75.6 | USA |
| 1 | USA | 1994 | 26670 | 2.607442 | 22.61822 | | | | | 26.08295 | United Stat | 75.77 | USA |
| 1 | USA | 1995 | 27574 | 2.80542 | 24.27398 | 44.6 | | | | 26.29285 | United Stat | 75.89 | USA |
| 1 | USA | 1996 | 28814 | 2.931204 | 24.58247 | | | | | 25.7854 | United Stat | 76.21 | USA |
| 1 | USA | 1997 | 30262 | 2.33769 | 25.59154 | | | | | 25.38429 | United Stat | 76.53 | USA |
| 1 | USA | 1998 | 31519 | 1.552279 | 24.69057 | | | | | 25.01884 | United Stat | 76.7 | USA |
| 1 | USA | 1999 | 33028 | 2.188027 | | | | | | 24.72505 | United Stat | 76.72 | USA |
| 1 | USA | 2000 | 34603 | 3.376857 | | 39.6 | | | | 24.44349 | United Stat | 76.86 | USA |
| 1 | USA | 2001 | 35341 | 2.826171 | | | | | | 23.04858 | United Stat | 76.97 | USA |
| 1 | USA | 2002 | 36180 | 1.586032 | | | | | | | United Stat | 77.04 | USA |
| 1 | USA | 2003 | 37548 | 2.270095 | | | | | | | United Stat | 77.2 | USA |

| | | | | | | | | | | | | | |
|---|---|---|---|---|---|---|---|---|---|---|---|---|---|
| 19 | Spain | 1960 | 1074 | | 16.24982 | 3.9 | | | | | Spain | 69.22 | Spain |
| 19 | Spain | 1961 | 1201 | 2.005919 | 17.6388 | | | | | | Spain | 69.6 | Spain |
| 19 | Spain | 1962 | 1335 | 5.634759 | 19.60613 | | | | | | Spain | 69.63 | Spain |
| 19 | Spain | 1963 | 1467 | 8.804627 | 19.67972 | | | | | | Spain | 69.79 | Spain |
| 19 | Spain | 1964 | 1554 | 6.950187 | 21.49239 | | | | | | Spain | 70.51 | Spain |
| 19 | Spain | 1965 | 1678 | 13.23638 | 22.70879 | 4.6 | | | | | Spain | 70.93 | Spain |
| 19 | Spain | 1966 | 1841 | 6.194115 | 23.89032 | | | | | | Spain | 71.17 | Spain |
| 19 | Spain | 1967 | 1954 | 6.414575 | 21.74692 | | | | | | Spain | 71.36 | Spain |
| 19 | Spain | 1968 | 2160 | 4.963315 | 24.6574 | | | | | | Spain | 71.65 | Spain |
| 19 | Spain | 1969 | 2447 | 2.124452 | 26.03309 | | | | | | Spain | 71.16 | Spain |
| 19 | Spain | 1970 | 2715 | 5.757617 | 27.45076 | 5.8 | | | | | Spain | 72.14 | Spain |
| 19 | Spain | 1971 | 2951 | 8.236041 | 27.57413 | | | | | 40.66722 | Spain | 71.73 | Spain |
| 19 | Spain | 1972 | 3293 | 8.289366 | 28.92659 | | | | | 41.69329 | Spain | 72.94 | Spain |
| 19 | Spain | 1973 | 3710 | 11.39021 | 29.88689 | | | | | 42.23267 | Spain | 72.72 | Spain |
| 19 | Spain | 1974 | 4227 | 15.71734 | 33.60297 | | | | | 43.1723 | Spain | 73.1 | Spain |
| 19 | Spain | 1975 | 4600 | 16.92566 | 30.8514 | 8.2 | | | | 42.08322 | Spain | 73.48 | Spain |
| 19 | Spain | 1976 | 4965 | 17.62931 | 31.89063 | | | | | 41.38425 | Spain | 73.8 | Spain |
| 19 | Spain | 1977 | 5364 | 24.52669 | 30.97939 | | | | | 40.52181 | Spain | 74.29 | Spain |
| 19 | Spain | 1978 | 5761 | 19.78421 | 29.52087 | | | | | 39.43257 | Spain | 74.48 | Spain |
| 19 | Spain | 1979 | 6188 | 15.66901 | 29.62632 | | | | | 38.96561 | Spain | 74.98 | Spain |
| 19 | Spain | 1980 | 6856 | 15.54989 | 33.52295 | 13.3 | 0.25176 | 0.31324 | 24.568 | 38.56755 | Spain | 75.52 | Spain |
| 19 | Spain | 1981 | 7447 | 14.55704 | 37.42521 | | | | | 37.67302 | Spain | 75.69 | Spain |
| 19 | Spain | 1982 | 7957 | 14.40796 | 38.38861 | | | | | 37.06181 | Spain | 76.31 | Spain |
| 19 | Spain | 1983 | 8378 | 12.17745 | 41.89354 | | | | | 36.7945 | Spain | 76.09 | Spain |
| 19 | Spain | 1984 | 8812 | 11.27433 | 43.44701 | | | | | 35.9208 | Spain | 76.47 | Spain |
| 19 | Spain | 1985 | 9259 | 8.817527 | 43.49968 | 17.6 | | | | 36.20907 | Spain | 76.4 | Spain |
| 19 | Spain | 1986 | 9744 | 8.796503 | 37.57776 | | | | | 36.6543 | Spain | 76.7 | Spain |
| 19 | Spain | 1987 | 10542 | 5.246259 | 38.54278 | | | | | 36.09407 | Spain | 76.95 | Spain |
| 19 | Spain | 1988 | 11434 | 4.840479 | 38.83916 | | | | | 35.9314 | Spain | 76.94 | Spain |
| 19 | Spain | 1989 | 12417 | 6.791468 | 39.45245 | | | | | 36.21816 | Spain | 76.99 | Spain |
| 19 | Spain | 1990 | 13365 | 6.71794 | 37.50269 | 25.5 | | | | 35.47665 | Spain | 76.99 | Spain |
| 19 | Spain | 1991 | 14152 | 5.935327 | 37.40708 | | | | | 34.68851 | Spain | 77.12 | Spain |
| 19 | Spain | 1992 | 14564 | 5.925724 | 38.03965 | | | | | 32.8619 | Spain | 77.58 | Spain |
| 19 | Spain | 1993 | 14699 | 4.568636 | 39.40948 | | | | | 31.16821 | Spain | 77.72 | Spain |
| 19 | Spain | 1994 | 15325 | 4.718468 | 44.39644 | | | | | 30.55055 | Spain | 78.1 | Spain |
| 19 | Spain | 1995 | 16032 | 4.674753 | 47.8529 | 28.6 | | | | 30.98291 | Spain | 78.19 | Spain |
| 19 | Spain | 1996 | 16720 | 3.558506 | 50.0733 | | | | | 30.45867 | Spain | 78.34 | Spain |
| 19 | Spain | 1997 | 17420 | 1.970708 | 55.55865 | | | | | 30.4414 | Spain | 78.84 | Spain |
| 19 | Spain | 1998 | 18479 | 1.833537 | 57.1253 | | | | | 30.19667 | Spain | 78.94 | Spain |
| 19 | Spain | 1999 | 19817 | 2.310672 | | | | | | 29.9892 | Spain | 78.95 | Spain |
| 19 | Spain | 2000 | 21074 | 3.432614 | | 30.7 | | | | 30.1937 | Spain | 79.42 | Spain |
| 19 | Spain | 2001 | 22257 | 3.591101 | | | | | | 29.92884 | Spain | 79.74 | Spain |
| 19 | Spain | 2002 | 23756 | 3.066777 | | | | | | 29.68476 | Spain | 79.83 | Spain |
| 19 | Spain | 2003 | 24812 | 3.033474 | | | | | | 29.58652 | Spain | 79.76 | Spain |
| 12 | Switzerlanc | 1960 | 3387 | | 53.2535 | 21.8 | | | | | Switzerlanc | 71.41 | Switzerland |
| 12 | Switzerlanc | 1961 | 3625 | 1.845371 | 54.59318 | | | | | | Switzerlanc | 71.74 | Switzerland |
| 12 | Switzerlanc | 1962 | 3788 | 4.315616 | 54.49474 | | | | | | Switzerlanc | 71.3 | Switzerland |
| 12 | Switzerlanc | 1963 | 3946 | 3.439719 | 53.69223 | | | | | | Switzerlanc | 71.28 | Switzerland |
| 12 | Switzerlanc | 1964 | 4158 | 3.081314 | 53.66071 | | | | | | Switzerlanc | 72.18 | Switzerland |
| 12 | Switzerlanc | 1965 | 4360 | 3.414517 | 55.15859 | 22.3 | | | | | Switzerlanc | 72.31 | Switzerland |
| 12 | Switzerlanc | 1966 | 4575 | 4.776131 | 55.51873 | | | | | | Switzerlanc | 72.44 | Switzerland |
| 12 | Switzerlanc | 1967 | 4790 | 4.021478 | 54.6139 | | | | | | Switzerlanc | 72.74 | Switzerland |
| 12 | Switzerlanc | 1968 | 5138 | 2.412351 | 56.8371 | | | | | | Switzerlanc | 72.7 | Switzerland |
| 12 | Switzerlanc | 1969 | 5645 | 2.489007 | 60.69416 | | | | | | Switzerlanc | 72.71 | Switzerland |
| 12 | Switzerlanc | 1970 | 6382 | 3.616027 | 63.58904 | 38 | | | | 36.26636 | Switzerlanc | 73.12 | Switzerland |
| 12 | Switzerlanc | 1971 | 6938 | 6.573013 | 60.33389 | | | | | 35.98696 | Switzerlanc | 73.23 | Switzerland |
| 12 | Switzerlanc | 1972 | 7406 | 6.660192 | 58.81929 | | | | | 35.70756 | Switzerlanc | 73.75 | Switzerland |
| 12 | Switzerlanc | 1973 | 7998 | 8.755041 | 59.54798 | | | | | 35.42816 | Switzerlanc | 74.06 | Switzerland |
| 12 | Switzerlanc | 1974 | 8800 | 9.766747 | 63.37842 | | | | | 35.14877 | Switzerlanc | 74.4 | Switzerland |
| 12 | Switzerlanc | 1975 | 8934 | 6.696453 | 56.72313 | 36.4 | | | | 34.77623 | Switzerlanc | 74.78 | Switzerland |
| 12 | Switzerlanc | 1976 | 9368 | 1.715862 | 60.43211 | | | | | 34.49683 | Switzerlanc | 74.91 | Switzerland |
| 12 | Switzerlanc | 1977 | 10240 | 1.28527 | 66.90281 | | | | | 34.21744 | Switzerlanc | 75.36 | Switzerland |
| 12 | Switzerlanc | 1978 | 11003 | 1.055814 | 64.06974 | | | | | 33.93804 | Switzerlanc | 75.3 | Switzerland |
| 12 | Switzerlanc | 1979 | 12189 | 3.647643 | 67.3141 | | | | | 33.65864 | Switzerlanc | 75.6 | Switzerland |
| 12 | Switzerlanc | 1980 | 13853 | 4.022026 | 72.83212 | 54.3 | 0.33524 | 0.33744 | 30.964 | 32.87 | Switzerlanc | 75.58 | Switzerland |
| 12 | Switzerlanc | 1981 | 15338 | 6.490219 | 72.36987 | | | | | 32.69 | Switzerlanc | 75.81 | Switzerland |
| 12 | Switzerlanc | 1982 | 15963 | 5.655212 | 67.87426 | | | | | 32.2 | Switzerlanc | 76.16 | Switzerland |
| 12 | Switzerlanc | 1983 | 16611 | 2.967687 | 68.34943 | | | | | 31.9 | Switzerlanc | 76.15 | Switzerland |
| 12 | Switzerlanc | 1984 | 17710 | 2.911208 | 72.16822 | | | | | 31.92 | Switzerlanc | 76.75 | Switzerland |
| 12 | Switzerlanc | 1985 | 18812 | 3.429986 | 74.65241 | 53.6 | | | | 32.01 | Switzerlanc | 76.87 | Switzerland |
| 12 | Switzerlanc | 1986 | 19458 | 0.740741 | 70.62199 | | | | | 31.89 | Switzerlanc | 77.03 | Switzerland |
| 12 | Switzerlanc | 1987 | 20120 | 1.447964 | 69.58219 | | | | | 31.46 | Switzerlanc | 77.34 | Switzerland |
| 12 | Switzerlanc | 1988 | 21334 | 1.884478 | 71.42004 | | | | | 31.61 | Switzerlanc | 77.36 | Switzerland |
| 12 | Switzerlanc | 1989 | 22965 | 3.162964 | 75.54489 | | | | | 31.69 | Switzerlanc | 77.56 | Switzerland |
| 12 | Switzerlanc | 1990 | 24518 | 5.37874 | 72.00161 | 53.8 | | | | 31.34 | Switzerlanc | 77.37 | Switzerland |
| 12 | Switzerlanc | 1991 | 24840 | 5.879392 | 68.58638 | | | | | 30.79 | Switzerlanc | 77.64 | Switzerland |
| 12 | Switzerlanc | 1992 | 25141 | 4.041076 | 67.86861 | | | | | 29.99 | Switzerlanc | 77.94 | Switzerland |
| 12 | Switzerlanc | 1993 | 25427 | 3.271797 | 66.71021 | | | | | 29.58 | Switzerlanc | 78.23 | Switzerland |
| 12 | Switzerlanc | 1994 | 26031 | 0.858407 | 66.58563 | | | | | 30.41 | Switzerlanc | 78.5 | Switzerland |
| 12 | Switzerlanc | 1995 | 26485 | 1.798719 | 66.15726 | 54.8 | | | | 29.83 | Switzerlanc | 78.56 | Switzerland |
| 12 | Switzerlanc | 1996 | 26394 | 0.818824 | 67.94028 | | | | | 28.81 | Switzerlanc | 79.05 | Switzerland |
| 12 | Switzerlanc | 1997 | 27850 | 0.521501 | 75.34065 | | | | | 28.19 | Switzerlanc | 79.23 | Switzerland |
| 12 | Switzerlanc | 1998 | 28835 | 0.01701 | | | | | | 27.43 | Switzerlanc | 79.47 | Switzerland |
| 12 | Switzerlanc | 1999 | 28887 | 0.82483 | | | | | | 27.43 | Switzerlanc | 79.73 | Switzerland |
| 12 | Switzerlanc | 2000 | 30461 | 1.543392 | | 55 | | | | 26.76 | Switzerlanc | 79.84 | Switzerland |
| 12 | Switzerlanc | 2001 | 30806 | 0.988372 | | | | | | 27.35 | Switzerlanc | 80.26 | Switzerland |
| 12 | Switzerlanc | 2002 | 32751 | 0.6415 | | | | | | 26.78 | Switzerlanc | 80.44 | Switzerland |
| 12 | Switzerlanc | 2003 | 33516 | 0.629239 | | | | | | 26.45 | Switzerlanc | 80.54 | Switzerland |

| | | | | | | | | | | | | | |
|---|---|---|---|---|---|---|---|---|---|---|---|---|---|
| 10 | Norway | 1960 | 1713 | | 73.68096 | 21.3 | | | | | Norway | 73.57 | Norway |
| 10 | Norway | 1961 | 1823 | 2.451108 | 72.13998 | | | | | | Norway | 73.57 | Norway |
| 10 | Norway | 1962 | 1905 | 5.268516 | 69.34479 | | | | | | Norway | 73.46 | Norway |
| 10 | Norway | 1963 | 1991 | 2.587041 | 70.4398 | | | | | | Norway | 73.1 | Norway |
| 10 | Norway | 1964 | 2112 | 5.679944 | 71.78108 | | | | | | Norway | 73.61 | Norway |
| 10 | Norway | 1965 | 2268 | 4.25959 | 71.81725 | 22.4 | | | | | Norway | 73.72 | Norway |
| 10 | Norway | 1966 | 2417 | 3.251337 | 72.5338 | | | | | | Norway | 74.01 | Norway |
| 10 | Norway | 1967 | 2620 | 4.433395 | 74.77306 | | | | | | Norway | 74.08 | Norway |
| 10 | Norway | 1968 | 2784 | 3.471534 | 73.59362 | | | | | | Norway | 73.95 | Norway |
| 10 | Norway | 1969 | 3035 | 3.067485 | 72.03036 | | | | | | Norway | 73.66 | Norway |
| 10 | Norway | 1970 | 3213 | 10.56548 | 74.13993 | 31.1 | | | | | Norway | 74.07 | Norway |
| 10 | Norway | 1971 | 3523 | 6.258412 | 73.00291 | | | | | 31.94468 | Norway | 74.17 | Norway |
| 10 | Norway | 1972 | 3814 | 7.219759 | 70.42838 | | | | | 32.23043 | Norway | 74.34 | Norway |
| 10 | Norway | 1973 | 4138 | 7.44241 | 76.50484 | | | | | 32.27221 | Norway | 74.44 | Norway |
| 10 | Norway | 1974 | 4728 | 9.414514 | 83.27903 | | | | | 33.08653 | Norway | 74.76 | Norway |
| 10 | Norway | 1975 | 5458 | 11.70707 | 78.7672 | 34.8 | | | | 34.62412 | Norway | 74.82 | Norway |
| 10 | Norway | 1976 | 6116 | 9.175756 | 79.93233 | | | | | 34.2097 | Norway | 75.05 | Norway |
| 10 | Norway | 1977 | 6742 | 9.043156 | 78.69983 | | | | | 34.0764 | Norway | 75.39 | Norway |
| 10 | Norway | 1978 | 7412 | 8.151506 | 72.255 | | | | | 35.43352 | Norway | 75.43 | Norway |
| 10 | Norway | 1979 | 8354 | 4.759825 | 77.27374 | | | | | 38.27974 | Norway | 75.41 | Norway |
| 10 | Norway | 1980 | 9557 | 10.89621 | 80.33016 | 39.3 | 0.33696 | 0.37418 | 31.594 | 40.38873 | Norway | 75.67 | Norway |
| 10 | Norway | 1981 | 10548 | 13.63705 | 79.6489 | | | | | 40.68092 | Norway | 75.88 | Norway |
| 10 | Norway | 1982 | 11205 | 11.37206 | 78.18887 | | | | | 40.84821 | Norway | 76.02 | Norway |
| 10 | Norway | 1983 | 12022 | 8.411048 | 76.31546 | | | | | 42.04797 | Norway | 76.08 | Norway |
| 10 | Norway | 1984 | 13220 | 6.279108 | 77.48017 | | | | | 43.46854 | Norway | 76.25 | Norway |
| 10 | Norway | 1985 | 14354 | 5.665824 | 78.74471 | 41 | | | | 42.85285 | Norway | 75.93 | Norway |
| 10 | Norway | 1986 | 15184 | 7.186768 | 72.85357 | | | | | 36.27047 | Norway | 76.25 | Norway |
| 10 | Norway | 1987 | 15823 | 8.725932 | 67.69511 | | | | | 35.80692 | Norway | 76.1 | Norway |
| 10 | Norway | 1988 | 16299 | 6.698484 | 67.40089 | | | | | 34.0042 | Norway | 76.26 | Norway |
| 10 | Norway | 1989 | 17043 | 4.555442 | 73.29363 | | | | | 35.5254 | Norway | 76.54 | Norway |
| 10 | Norway | 1990 | 18004 | 4.113033 | 74.73464 | 69.6 | | | | 35.66044 | Norway | 76.58 | Norway |
| 10 | Norway | 1991 | 19212 | 3.420683 | 72.62285 | | | | | 34.55202 | Norway | 77.04 | Norway |
| 10 | Norway | 1992 | 20185 | 2.342116 | 69.54725 | | | | | 34.17055 | Norway | 77.24 | Norway |
| 10 | Norway | 1993 | 21088 | 2.271489 | 70.1258 | | | | | 33.76404 | Norway | 77.22 | Norway |
| 10 | Norway | 1994 | 22534 | 1.398555 | 70.58623 | | | | | 34.25858 | Norway | 77.78 | Norway |
| 10 | Norway | 1995 | 23874 | 2.456405 | 70.1032 | 66.7 | | | | 35.35278 | Norway | 77.79 | Norway |
| 10 | Norway | 1996 | 26263 | 1.258694 | 72.61958 | | | | | 37.7201 | Norway | 78.23 | Norway |
| 10 | Norway | 1997 | 27776 | 2.580741 | 75.46217 | | | | | 38.2629 | Norway | 78.23 | Norway |
| 10 | Norway | 1998 | 27294 | 2.255686 | 74.28209 | | | | | 33.82029 | Norway | 78.41 | Norway |
| 10 | Norway | 1999 | 30011 | 2.333139 | | | | | | 35.71146 | Norway | 78.38 | Norway |
| 10 | Norway | 2000 | 36273 | 3.086068 | | 62.5 | | | | 42.95295 | Norway | 78.69 | Norway |
| 10 | Norway | 2001 | 37078 | 3.017378 | | | | | | 40.8742 | Norway | 78.89 | Norway |
| 10 | Norway | 2002 | 36617 | 1.288146 | | | | | | 37.90837 | Norway | 78.96 | Norway |
| 10 | Norway | 2003 | 37245 | 2.475397 | | | | | | 37.51624 | Norway | 79.53 | Norway |
| 18 | Portugal | 1960 | 728 | | 37.35394 | 3.5 | | | | | Portugal | 64.17 | Portugal |
| 18 | Portugal | 1961 | 783 | 1.549002 | 39.98289 | | | | | | Portugal | 62.78 | Portugal |
| 18 | Portugal | 1962 | 848 | 2.662071 | 38.32179 | | | | | | Portugal | 64.3 | Portugal |
| 18 | Portugal | 1963 | 905 | 2.021573 | 39.37737 | | | | | | Portugal | 64.93 | Portugal |
| 18 | Portugal | 1964 | 979 | 3.4 | 50.41978 | | | | | | Portugal | 65.16 | Portugal |
| 18 | Portugal | 1965 | 1084 | 3.481625 | 52.90274 | 3.9 | | | | | Portugal | 66.09 | Portugal |
| 18 | Portugal | 1966 | 1170 | 5.046729 | 52.80592 | | | | | | Portugal | 65.6 | Portugal |
| 18 | Portugal | 1967 | 1293 | 5.516014 | 51.5496 | | | | | | Portugal | 66.5 | Portugal |
| 18 | Portugal | 1968 | 1475 | 6.070827 | 49.79484 | | | | | | Portugal | 66.8 | Portugal |
| 18 | Portugal | 1969 | 1592 | 7.402512 | 48.17641 | | | | | | Portugal | 66.4 | Portugal |
| 18 | Portugal | 1970 | 1916 | 4.533333 | 50.21158 | 6.8 | | | | | Portugal | 67.06 | Portugal |
| 18 | Portugal | 1971 | 2153 | 7.496075 | 51.90705 | | | | | 35.13776 | Portugal | 66.85 | Portugal |
| 18 | Portugal | 1972 | 2420 | 8.94487 | 53.73326 | | | | | 35.94559 | Portugal | 69.17 | Portugal |
| 18 | Portugal | 1973 | 2845 | 10.35523 | 54.88761 | | | | | 35.96346 | Portugal | 68.58 | Portugal |
| 18 | Portugal | 1974 | 3048 | 27.96842 | 62.63343 | | | | | 37.54448 | Portugal | 69.13 | Portugal |
| 18 | Portugal | 1975 | 3045 | 20.40816 | 48.22217 | 9.6 | | | | 35.2115 | Portugal | 68.86 | Portugal |
| 18 | Portugal | 1976 | 3407 | 18.2499 | 43.77534 | | | | | 34.72538 | Portugal | 69.08 | Portugal |
| 18 | Portugal | 1977 | 3784 | 27.19167 | 47.03732 | | | | | 34.72417 | Portugal | 70.33 | Portugal |
| 18 | Portugal | 1978 | 4120 | 22.64299 | 47.7536 | | | | | 33.15321 | Portugal | 70.79 | Portugal |
| 18 | Portugal | 1979 | 4663 | 23.53758 | 58.92996 | | | | | 31.94781 | Portugal | 71.59 | Portugal |
| 18 | Portugal | 1980 | 5263 | 16.69189 | 62.97908 | 10.6 | 0.24082 | 0.29798 | 26.488 | 32.87777 | Portugal | 71.66 | Portugal |
| 18 | Portugal | 1981 | 5812 | 20.04076 | 64.51057 | | | | | 33.93985 | Portugal | 71.89 | Portugal |
| 18 | Portugal | 1982 | 6263 | 22.73022 | 64.73094 | | | | | 33.64522 | Portugal | 72.7 | Portugal |
| 18 | Portugal | 1983 | 6479 | 25.10564 | 68.44718 | | | | | 34.49608 | Portugal | 72.64 | Portugal |
| 18 | Portugal | 1984 | 6570 | 28.78333 | 74.85384 | | | | | 32.84181 | Portugal | 72.93 | Portugal |
| 18 | Portugal | 1985 | 6959 | 19.6454 | 71.50999 | 13.7 | | | | 32.82258 | Portugal | 73.21 | Portugal |
| 18 | Portugal | 1986 | 7414 | 11.76311 | 62.80074 | | | | | 30.64454 | Portugal | 73.64 | Portugal |
| 18 | Portugal | 1987 | 8126 | 9.343148 | 69.23901 | | | | | 30.37263 | Portugal | 74.05 | Portugal |
| 18 | Portugal | 1988 | 9057 | 9.697048 | 73.42918 | | | | | 32.47451 | Portugal | 74.09 | Portugal |
| 18 | Portugal | 1989 | 10042 | 12.61655 | 75.04156 | | | | | 32.34439 | Portugal | 74.66 | Portugal |
| 18 | Portugal | 1990 | 10894 | 13.37246 | 75.17775 | 14.8 | | | | 31.71348 | Portugal | 74.29 | Portugal |
| 18 | Portugal | 1991 | 11783 | 10.92623 | 69.90818 | | | | | 30.28444 | Portugal | 74.23 | Portugal |
| 18 | Portugal | 1992 | 12219 | 8.941667 | 65.14225 | | | | | 30.2792 | Portugal | 74.87 | Portugal |
| 18 | Portugal | 1993 | 12236 | 6.501951 | 62.44869 | | | | | 29.60257 | Portugal | 74.75 | Portugal |
| 18 | Portugal | 1994 | 12614 | 5.214394 | 65.60572 | | | | | 30.64367 | Portugal | 75.72 | Portugal |
| 18 | Portugal | 1995 | 13413 | 4.123148 | 68.57445 | 14.7 | | | | 31.49808 | Portugal | 75.54 | Portugal |
| 18 | Portugal | 1996 | 13974 | 3.120697 | 69.22182 | | | | | 31.74154 | Portugal | 75.47 | Portugal |
| 18 | Portugal | 1997 | 14804 | 2.161612 | 71.54989 | | | | | 31.63949 | Portugal | 75.91 | Portugal |
| 18 | Portugal | 1998 | 15401 | 2.71644 | | | | | | 31.13629 | Portugal | 76.11 | Portugal |
| 18 | Portugal | 1999 | 16363 | 2.303886 | | | | | | 30.41585 | Portugal | 76.31 | Portugal |
| 18 | Portugal | 2000 | 17353 | 2.846721 | | 14.9 | | | | 29.99342 | Portugal | 76.81 | Portugal |
| 18 | Portugal | 2001 | 18071 | 4.394757 | | | | | | 29.39245 | Portugal | 77.13 | Portugal |
| 18 | Portugal | 2002 | 18799 | 3.545052 | | | | | | 28.68818 | Portugal | 77.35 | Portugal |
| 18 | Portugal | 2003 | 17603 | 3.283333 | | | | | | | Portugal | 77.55 | Portugal |

| | | | | | | | | | | | | | |
|---|---|---|---|---|---|---|---|---|---|---|---|---|---|
| 9 | Netherland | 1960 | 2113 | | 90.88709 | 11.1 | | | | | Netherland | 73.36 | Netherlands |
| 9 | Netherland | 1961 | 2111 | 1.732093 | 88.21291 | | | | | | Netherland | 73.55 | Netherlands |
| 9 | Netherland | 1962 | 2275 | 2.286038 | 86.70377 | | | | | | Netherland | 73.22 | Netherlands |
| 9 | Netherland | 1963 | 2360 | 3.343184 | 87.91217 | | | | | | Netherland | 73.35 | Netherlands |
| 9 | Netherland | 1964 | 2566 | 5.790884 | 86.53982 | | | | | | Netherland | 73.73 | Netherlands |
| 9 | Netherland | 1965 | 2739 | 5.794898 | 83.92977 | 13.6 | | | | | Netherland | 73.6 | Netherlands |
| 9 | Netherland | 1966 | 2874 | 5.79687 | 82.38142 | | | | | | Netherland | 73.55 | Netherlands |
| 9 | Netherland | 1967 | 3078 | 3.486793 | 79.59118 | | | | | | Netherland | 73.83 | Netherlands |
| 9 | Netherland | 1968 | 3399 | 3.675613 | 79.65446 | | | | | | Netherland | 73.64 | Netherlands |
| 9 | Netherland | 1969 | 3951 | 7.42825 | 82.78465 | | | | | | Netherland | 73.56 | Netherlands |
| 9 | Netherland | 1970 | 4342 | 3.679937 | 88.73793 | 45.8 | | | | | Netherland | 73.62 | Netherlands |
| 9 | Netherland | 1971 | 4705 | 7.47758 | 88.53603 | | | | | 37.20113 | Netherland | 73.86 | Netherlands |
| 9 | Netherland | 1972 | 4984 | 7.79175 | 84.7172 | | | | | 36.9602 | Netherland | 73.78 | Netherlands |
| 9 | Netherland | 1973 | 5475 | 8.013519 | 88.92212 | | | | | 36.77002 | Netherland | 74.22 | Netherlands |
| 9 | Netherland | 1974 | 6165 | 9.609367 | 102.1402 | | | | | 36.93427 | Netherland | 74.61 | Netherlands |
| 9 | Netherland | 1975 | 6699 | 10.21273 | 93.63818 | 44.3 | | | | 35.57053 | Netherland | 74.55 | Netherlands |
| 9 | Netherland | 1976 | 7345 | 9.057487 | 95.7662 | | | | | 35.76351 | Netherland | 74.67 | Netherlands |
| 9 | Netherland | 1977 | 7959 | 6.474103 | 91.19662 | | | | | 35.20782 | Netherland | 75.26 | Netherlands |
| 9 | Netherland | 1978 | 8676 | 4.080017 | 87.71375 | | | | | 34.13189 | Netherland | 75.17 | Netherlands |
| 9 | Netherland | 1979 | 9504 | 4.210454 | 96.2868 | | | | | 34.08447 | Netherland | 75.65 | Netherlands |
| 9 | Netherland | 1980 | 10458 | 6.73332 | 102.6575 | 44.1 | 0.29368 | 0.30778 | 21.84 | 34.51798 | Netherland | 75.77 | Netherlands |
| 9 | Netherland | 1981 | 11304 | 6.748466 | 109.6119 | | | | | 34.74647 | Netherland | 75.98 | Netherlands |
| 9 | Netherland | 1982 | 11784 | 5.862069 | 107.3393 | | | | | 34.06893 | Netherland | 76.06 | Netherlands |
| 9 | Netherland | 1983 | 12419 | 2.823019 | 106.9498 | | | | | 33.90657 | Netherland | 76.25 | Netherlands |
| 9 | Netherland | 1984 | 13234 | 3.273495 | 114.4047 | | | | | 34.55178 | Netherland | 76.32 | Netherlands |
| 9 | Netherland | 1985 | 13938 | 2.223926 | 116.7998 | 44.7 | | | | 34.86816 | Netherland | 76.37 | Netherlands |
| 9 | Netherland | 1986 | 14613 | 0.100025 | 97.60883 | | | | | 32.41933 | Netherland | 76.34 | Netherlands |
| 9 | Netherland | 1987 | 15197 | -0.7078 | 96.81971 | | | | | 30.76184 | Netherland | 76.81 | Netherlands |
| 9 | Netherland | 1988 | 16082 | 0.746394 | 101.0815 | | | | | 30.96866 | Netherland | 76.99 | Netherlands |
| 9 | Netherland | 1989 | 17387 | 1.082161 | 106.4914 | | | | | 30.66598 | Netherland | 76.83 | Netherlands |
| 9 | Netherland | 1990 | 18665 | 3.285469 | 103.6821 | 45.4 | | | | 30.71949 | Netherland | 77 | Netherlands |
| 9 | Netherland | 1991 | 19626 | 3.133333 | 103.301 | | | | | 30.41988 | Netherland | 77.15 | Netherlands |
| 9 | Netherland | 1992 | 20224 | 3.183581 | 99.63787 | | | | | 29.34567 | Netherland | 77.36 | Netherlands |
| 9 | Netherland | 1993 | 20679 | 2.584182 | 94.70643 | | | | | 28.65413 | Netherland | 77.04 | Netherlands |
| 9 | Netherland | 1994 | 21588 | 2.801527 | 95.98548 | | | | | 28.54458 | Netherland | 77.51 | Netherlands |
| 9 | Netherland | 1995 | 22585 | 2.095282 | 99.49503 | 46.3 | | | | 28.74623 | Netherland | 77.54 | Netherlands |
| 9 | Netherland | 1996 | 23531 | 2.016667 | 101.0114 | | | | | 28.43318 | Netherland | 77.55 | Netherlands |
| 9 | Netherland | 1997 | 24692 | 2.15651 | 104.8999 | | | | | 27.83015 | Netherland | 77.92 | Netherlands |
| 9 | Netherland | 1998 | 25811 | 1.983048 | | | | | | 27.106 | Netherland | 78 | Netherlands |
| 9 | Netherland | 1999 | 26654 | 2.211071 | | | | | | 26.31304 | Netherland | 77.96 | Netherlands |
| 9 | Netherland | 2000 | 28467 | 2.52378 | | 45.4 | | | | 26.79898 | Netherland | 78.12 | Netherlands |
| 9 | Netherland | 2001 | 30359 | 4.534231 | | | | | | 26.62816 | Netherland | 78.33 | Netherlands |
| 9 | Netherland | 2002 | 31284 | 3.467068 | | | | | | 25.82768 | Netherland | 78.41 | Netherlands |
| 9 | Netherland | 2003 | 31792 | 2.11464 | | | | | | | Netherland | 78.66 | Netherlands |
| 21 | New Zeala | 1960 | 2545 | | 52.54882 | 58 | | | | | New Zeala | 71.22 | New Zealand |
| 21 | New Zeala | 1961 | 2659 | 1.830283 | 52.94457 | | | | | | New Zeala | 70.93 | New Zealand |
| 21 | New Zeala | 1962 | 2713 | 2.696079 | 48.80368 | | | | | | New Zeala | 71.2 | New Zealand |
| 21 | New Zeala | 1963 | 2866 | 1.909308 | 53.84496 | | | | | | New Zeala | 71.27 | New Zealand |
| 21 | New Zeala | 1964 | 3001 | 3.434817 | 50.79603 | | | | | | New Zeala | 71.29 | New Zealand |
| 21 | New Zeala | 1965 | 3224 | 3.396226 | 44.78164 | 54.3 | | | | | New Zeala | 71.21 | New Zealand |
| 21 | New Zeala | 1966 | 3481 | 2.773723 | 45.96717 | | | | | | New Zeala | 71.07 | New Zealand |
| 21 | New Zeala | 1967 | 3360 | 6.107955 | 39.77977 | | | | | | New Zeala | 71.44 | New Zealand |
| 21 | New Zeala | 1968 | 3489 | 4.283802 | 44.66377 | | | | | | New Zeala | 71.09 | New Zealand |
| 21 | New Zeala | 1969 | 3969 | 4.878049 | 46.90852 | | | | | | New Zeala | 71.47 | New Zealand |
| 21 | New Zeala | 1970 | 4136 | 6.548347 | 48.32402 | 54.5 | | | | | New Zeala | 71.22 | New Zealand |
| 21 | New Zeala | 1971 | 4438 | 10.30303 | 45.48153 | | | | | 34.0294 | New Zeala | 71.72 | New Zealand |
| 21 | New Zeala | 1972 | 4780 | 6.776557 | 47.43124 | | | | | 33.20894 | New Zeala | 71.83 | New Zealand |
| 21 | New Zeala | 1973 | 5335 | 8.233276 | 49.57341 | | | | | 32.4518 | New Zeala | 71.68 | New Zealand |
| 21 | New Zeala | 1974 | 6041 | 11.25198 | 55.45852 | | | | | 35.5166 | New Zeala | 71.94 | New Zealand |
| 21 | New Zeala | 1975 | 6382 | 14.52991 | 54.88925 | 57.1 | | | | 34.52904 | New Zeala | 72.2 | New Zealand |
| 21 | New Zeala | 1976 | 6748 | 16.79104 | 57.88928 | | | | | 34.71202 | New Zeala | 72.4 | New Zealand |
| 21 | New Zeala | 1977 | 6867 | 14.58999 | 56.79647 | | | | | 32.95466 | New Zeala | 72.14 | New Zealand |
| 21 | New Zeala | 1978 | 7301 | 11.89591 | 55.04187 | | | | | 32.50108 | New Zeala | 73.01 | New Zealand |
| 21 | New Zeala | 1979 | 7915 | 13.70432 | 61.89754 | | | | | 32.65749 | New Zeala | 73.05 | New Zealand |
| 21 | New Zeala | 1980 | 8656 | 17.09277 | 62.08681 | 46.1 | 0.26664 | 0.29516 | 25.522 | 32.18576 | New Zeala | 72.87 | New Zealand |
| 21 | New Zeala | 1981 | 9736 | 15.34623 | 62.44667 | | | | | 34.01445 | New Zeala | 73.63 | New Zealand |
| 21 | New Zeala | 1982 | 10687 | 16.1709 | 62.35155 | | | | | 34.55593 | New Zeala | 73.73 | New Zealand |
| 21 | New Zeala | 1983 | 11262 | 7.35568 | 61.91337 | | | | | 33.40371 | New Zeala | 73.83 | New Zealand |
| 21 | New Zeala | 1984 | 12141 | 6.201214 | 70.57388 | | | | | 34.58643 | New Zeala | 74.37 | New Zealand |
| 21 | New Zeala | 1985 | 12556 | 15.39404 | 64.61287 | 46.6 | | | | 33.26814 | New Zeala | 73.87 | New Zealand |
| 21 | New Zeala | 1986 | 13085 | 13.21782 | 55.48104 | | | | | 31.91995 | New Zeala | 74.12 | New Zealand |
| 21 | New Zeala | 1987 | 13402 | 15.74115 | 52.39779 | | | | | 30.22941 | New Zeala | 74.21 | New Zealand |
| 21 | New Zeala | 1988 | 13569 | 6.375142 | 50.62526 | | | | | 28.58743 | New Zeala | 74.47 | New Zealand |
| 21 | New Zeala | 1989 | 14124 | 7.490603 | 53.81931 | | | | | 29.19099 | New Zeala | 74.89 | New Zealand |
| 21 | New Zeala | 1990 | 14420 | 5.506608 | 54.53501 | 24.1 | | | | 27.92192 | New Zeala | 75.43 | New Zealand |
| 21 | New Zeala | 1991 | 14252 | 1.670146 | 56.42813 | | | | | 27.3392 | New Zeala | 76.07 | New Zealand |
| 21 | New Zeala | 1992 | 14679 | 1.026694 | 61.14054 | | | | | 27.11838 | New Zeala | 76.19 | New Zealand |
| 21 | New Zeala | 1993 | 15605 | 1.422764 | 59.26334 | | | | | 27.06921 | New Zeala | 76.5 | New Zealand |
| 21 | New Zeala | 1994 | 16686 | 2.40481 | 60.43647 | | | | | 27.76848 | New Zeala | 76.92 | New Zealand |
| 21 | New Zeala | 1995 | 17402 | 3.739837 | 58.59611 | 26.6 | | | | 26.99032 | New Zeala | 76.79 | New Zealand |
| 21 | New Zeala | 1996 | 17879 | 2.298851 | 57.03107 | | | | | 26.74216 | New Zeala | 76.81 | New Zealand |
| 21 | New Zeala | 1997 | 18510 | 1.174668 | 57.1321 | | | | | 26.50679 | New Zeala | 77.36 | New Zealand |
| 21 | New Zeala | 1998 | 18669 | 1.287229 | | | | | | 25.27657 | New Zeala | 78.17 | New Zealand |
| 21 | New Zeala | 1999 | 19937 | -0.1246 | | | | | | 25.51298 | New Zeala | 77.92 | New Zealand |
| 21 | New Zeala | 2000 | 20789 | 2.619761 | | 26.3 | | | | 25.29168 | New Zeala | 78.67 | New Zealand |
| 21 | New Zeala | 2001 | 21825 | 2.625821 | | | | | | | New Zeala | 78.68 | New Zealand |
| 21 | New Zeala | 2002 | 22662 | 2.677091 | | | | | | | New Zeala | 78.88 | New Zealand |
| 21 | New Zeala | 2003 | 23728 | 1.753576 | | | | | | | New Zeala | 79.16 | New Zealand |

| | | | | | | | | | | | | | |
|---|---|---|---|---|---|---|---|---|---|---|---|---|---|
| 8 | Italy | 1960 | 1620 | | 26.57343 | 13.9 | | | | | Italy | 69.19 | Italy |
| 8 | Italy | 1961 | 1763 | 2.702703 | 26.88328 | | | | | | Italy | 69.83 | Italy |
| 8 | Italy | 1962 | 1900 | 3.947368 | 27.11724 | | | | | | Italy | 69.22 | Italy |
| 8 | Italy | 1963 | 2021 | 7.594937 | 27.78547 | | | | | | Italy | 69.32 | Italy |
| 8 | Italy | 1964 | 2093 | 5.882353 | 26.7552 | | | | | | Italy | 70.38 | Italy |
| 8 | Italy | 1965 | 2205 | 4.444445 | 27.67776 | 14.1 | | | | | Italy | 70.25 | Italy |
| 8 | Italy | 1966 | 2400 | 3.191489 | 29.03006 | | | | | | Italy | 70.99 | Italy |
| 8 | Italy | 1967 | 2624 | 3.092783 | 29.30026 | | | | | | Italy | 71.04 | Italy |
| 8 | Italy | 1968 | 2910 | 2 | 29.87667 | | | | | | Italy | 70.85 | Italy |
| 8 | Italy | 1969 | 3227 | 1.960784 | 31.84477 | | | | | | Italy | 70.87 | Italy |
| 8 | Italy | 1970 | 3602 | 4.807693 | 32.85419 | 17.5 | | | | | Italy | 71.63 | Italy |
| 8 | Italy | 1971 | 3834 | 5.504587 | 33.22466 | | | | | 40.38538 | Italy | 71.88 | Italy |
| 8 | Italy | 1972 | 4101 | 5.217391 | 34.73354 | | | | | 39.51487 | Italy | 72.15 | Italy |
| 8 | Italy | 1973 | 4594 | 10.7438 | 36.74186 | | | | | 40.68504 | Italy | 72.1 | Italy |
| 8 | Italy | 1974 | 5240 | 19.40298 | 44.55848 | | | | | 42.32259 | Italy | 72.81 | Italy |
| 8 | Italy | 1975 | 5601 | 16.875 | 41.32477 | 18.6 | | | | 41.31369 | Italy | 72.72 | Italy |
| 8 | Italy | 1976 | 6288 | 16.57754 | 45.4257 | | | | | 42.21162 | Italy | 73.07 | Italy |
| 8 | Italy | 1977 | 6821 | 17.43119 | 45.85133 | | | | | 41.67762 | Italy | 73.44 | Italy |
| 8 | Italy | 1978 | 7542 | 12.10938 | 45.11474 | | | | | 41.04507 | Italy | 73.78 | Italy |
| 8 | Italy | 1979 | 8594 | 14.63415 | 47.70631 | | | | | 40.67571 | Italy | 74.11 | Italy |
| 8 | Italy | 1980 | 9643 | 21.2766 | 46.6544 | 29.2 | 0.37278 | 0.32452 | 26.728 | 40.37956 | Italy | 74.06 | Italy |
| 8 | Italy | 1981 | 10593 | 17.79449 | 48.83625 | | | | | 39.03865 | Italy | 74.44 | Italy |
| 8 | Italy | 1982 | 11275 | 16.38298 | 47.1122 | | | | | 38.13298 | Italy | 74.92 | Italy |
| 8 | Italy | 1983 | 11812 | 14.62523 | 43.48412 | | | | | 36.95853 | Italy | 74.73 | Italy |
| 8 | Italy | 1984 | 12543 | 10.84529 | 45.80589 | | | | | 36.52842 | Italy | 75.49 | Italy |
| 8 | Italy | 1985 | 13285 | 9.208633 | 46.10847 | 30.3 | | | | 36.09994 | Italy | 75.6 | Italy |
| 8 | Italy | 1986 | 13896 | 5.797101 | 38.94103 | | | | | 34.897 | Italy | 75.91 | Italy |
| 8 | Italy | 1987 | 14688 | 4.732254 | 38.36825 | | | | | 34.5781 | Italy | 76.34 | Italy |
| 8 | Italy | 1988 | 15784 | 5.112961 | 37.96268 | | | | | 34.44107 | Italy | 76.52 | Italy |
| 8 | Italy | 1989 | 16875 | 6.221719 | 40.16734 | | | | | 34.55434 | Italy | 76.92 | Italy |
| 8 | Italy | 1990 | 17946 | 6.496273 | 40.0452 | 31.5 | | | | 33.898 | Italy | 76.99 | Italy |
| 8 | Italy | 1991 | 18845 | 6.3 | 37.98859 | | | | | 32.9108 | Italy | 76.99 | Italy |
| 8 | Italy | 1992 | 19367 | 5.079962 | 39.34541 | | | | | 32.38869 | Italy | 77.36 | Italy |
| 8 | Italy | 1993 | 19778 | 4.476276 | 42.43132 | | | | | 31.35425 | Italy | 77.65 | Italy |
| 8 | Italy | 1994 | 20577 | 4.027421 | 45.07429 | | | | | 31.30136 | Italy | 77.87 | Italy |
| 8 | Italy | 1995 | 21532 | 5.244371 | 51.2474 | 32.4 | | | | 31.50129 | Italy | 78.12 | Italy |
| 8 | Italy | 1996 | 22235 | 3.974524 | 48.17212 | | | | | 30.50213 | Italy | 78.44 | Italy |
| 8 | Italy | 1997 | 22810 | 2.043106 | 50.31563 | | | | | 30.06423 | Italy | 78.73 | Italy |
| 8 | Italy | 1998 | 23840 | 1.96294 | | | | | | 29.91003 | Italy | 78.82 | Italy |
| 8 | Italy | 1999 | 24402 | 1.655629 | | | | | | 29.31141 | Italy | 79.21 | Italy |
| 8 | Italy | 2000 | 25759 | 2.537687 | | 32 | | | | 29.04274 | Italy | 79.62 | Italy |
| 8 | Italy | 2001 | 26586 | 2.785166 | | | | | | 28.74293 | Italy | 79.89 | Italy |
| 8 | Italy | 2002 | 27320 | 2.46532 | | | | | | 28.28014 | Italy | 80.12 | Italy |
| 8 | Italy | 2003 | 27537 | 2.672559 | | | | | | 27.80482 | Italy | 80.19 | Italy |
| 14 | Japan | 1960 | 1010 | | 20.95434 | 30.9 | | | | | Japan | 67.7 | Japan |
| 14 | Japan | 1961 | 1143 | 5.404726 | 20.12309 | | | | | | Japan | 68.35 | Japan |
| 14 | Japan | 1962 | 1262 | 6.773193 | 18.6711 | | | | | | Japan | 68.63 | Japan |
| 14 | Japan | 1963 | 1374 | 7.66138 | 18.86199 | | | | | | Japan | 69.71 | Japan |
| 14 | Japan | 1964 | 1546 | 3.838174 | 19.13273 | | | | | | Japan | 70.18 | Japan |
| 14 | Japan | 1965 | 1647 | 6.593407 | 19.6008 | 36.7 | | | | | Japan | 70.23 | Japan |
| 14 | Japan | 1966 | 1889 | 5.079663 | 19.55724 | | | | | | Japan | 71.04 | Japan |
| 14 | Japan | 1967 | 2133 | 3.977881 | 19.05209 | | | | | | Japan | 71.34 | Japan |
| 14 | Japan | 1968 | 2496 | 5.369703 | 19.07467 | | | | | | Japan | 71.66 | Japan |
| 14 | Japan | 1969 | 2917 | 5.242592 | 19.4848 | | | | | | Japan | 71.89 | Japan |
| 14 | Japan | 1970 | 3293 | 7.673267 | 20.33049 | 33 | | | | | Japan | 71.99 | Japan |
| 14 | Japan | 1971 | 3575 | 6.416972 | 20.70205 | | | | | 45.17072 | Japan | 72.81 | Japan |
| 14 | Japan | 1972 | 3988 | 4.824022 | 18.85861 | | | | | 44.57824 | Japan | 73.24 | Japan |
| 14 | Japan | 1973 | 4491 | 11.64593 | 20.04692 | | | | | 45.35773 | Japan | 73.4 | Japan |
| 14 | Japan | 1974 | 4778 | 23.15457 | 27.94505 | | | | | 43.6329 | Japan | 73.83 | Japan |
| 14 | Japan | 1975 | 5300 | 11.78279 | 25.55243 | 38.1 | | | | 41.40548 | Japan | 74.34 | Japan |
| 14 | Japan | 1976 | 5768 | 9.349221 | 26.3121 | | | | | 41.4841 | Japan | 74.74 | Japan |
| 14 | Japan | 1977 | 6344 | 8.158704 | 24.55252 | | | | | 40.43694 | Japan | 75.31 | Japan |
| 14 | Japan | 1978 | 7084 | 4.210798 | 20.49978 | | | | | 40.69216 | Japan | 75.64 | Japan |
| 14 | Japan | 1979 | 8027 | 3.730788 | 24.03824 | | | | | 40.60047 | Japan | 76.15 | Japan |
| 14 | Japan | 1980 | 8931 | 7.778707 | 28.28039 | 39.9 | 0.31988 | 0.41958 | 34.986 | 41.00015 | Japan | 76.12 | Japan |
| 14 | Japan | 1981 | 9986 | 4.911308 | 28.64903 | | | | | 40.91642 | Japan | 76.54 | Japan |
| 14 | Japan | 1982 | 10813 | 2.736976 | 28.35613 | | | | | 40.32062 | Japan | 77.05 | Japan |
| 14 | Japan | 1983 | 11346 | 1.882329 | 26.09699 | | | | | 39.47266 | Japan | 77.09 | Japan |
| 14 | Japan | 1984 | 12064 | 2.27158 | 27.26116 | | | | | 39.83451 | Japan | 77.49 | Japan |
| 14 | Japan | 1985 | 12978 | 2.023692 | 25.54118 | 41.9 | | | | 39.59441 | Japan | 77.78 | Japan |
| 14 | Japan | 1986 | 13590 | 0.619255 | 18.74487 | | | | | 39.01246 | Japan | 78.2 | Japan |
| 14 | Japan | 1987 | 14425 | 0.125012 | 17.5562 | | | | | 38.94236 | Japan | 78.61 | Japan |
| 14 | Japan | 1988 | 15862 | 0.672301 | 17.79494 | | | | | 39.11177 | Japan | 78.53 | Japan |
| 14 | Japan | 1989 | 17269 | 2.280099 | 19.78006 | | | | | 39.04401 | Japan | 78.96 | Japan |
| 14 | Japan | 1990 | 18815 | 3.059416 | 20.64731 | 44.5 | | | | 39.36253 | Japan | 78.97 | Japan |
| 14 | Japan | 1991 | 20055 | 3.240112 | 18.73083 | | | | | 39.02355 | Japan | 79.24 | Japan |
| 14 | Japan | 1992 | 20648 | 1.72701 | 17.88268 | | | | | 37.85884 | Japan | 79.28 | Japan |
| 14 | Japan | 1993 | 21122 | 1.28404 | 16.31121 | | | | | 36.38006 | Japan | 79.43 | Japan |
| 14 | Japan | 1994 | 21757 | 0.706203 | 16.44124 | | | | | 34.92773 | Japan | 79.82 | Japan |
| 14 | Japan | 1995 | 22551 | -0.12673 | 17.31402 | 46.8 | | | | 34.21719 | Japan | 79.66 | Japan |
| 14 | Japan | 1996 | 23714 | 0.135352 | 19.33235 | | | | | 33.91785 | Japan | 80.35 | Japan |
| 14 | Japan | 1997 | 24478 | 1.731858 | 20.9999 | | | | | 33.71395 | Japan | 80.56 | Japan |
| 14 | Japan | 1998 | 24429 | 0.656037 | 21.14499 | | | | | 32.70455 | Japan | 80.63 | Japan |
| 14 | Japan | 1999 | 24709 | -0.33826 | | | | | | 32.42955 | Japan | 80.6 | Japan |
| 14 | Japan | 2000 | 26015 | -0.67053 | | 47.9 | | | | 32.23116 | Japan | 81.19 | Japan |
| 14 | Japan | 2001 | 26619 | -0.73339 | | | | | | 30.96192 | Japan | 81.53 | Japan |
| 14 | Japan | 2002 | 27196 | -0.91512 | | | | | | 30.39852 | Japan | 81.81 | Japan |
| 14 | Japan | 2003 | 28071 | -0.25419 | | | | | | | Japan | 81.87 | Japan |

| | | | | | | | | | | | | | |
|---|---|---|---|---|---|---|---|---|---|---|---|---|---|
| 6 | France | 1960 | 1858 | | 26.91962 | 25.7 | | | | | France | 70.37 | France |
| 6 | France | 1961 | 1959 | 2.414562 | 26.21155 | | | | | | France | 70.95 | France |
| 6 | France | 1962 | 2099 | 5.24725 | 24.89856 | | | | | | France | 70.47 | France |
| 6 | France | 1963 | 2205 | 4.93969 | 25.00591 | | | | | | France | 70.32 | France |
| 6 | France | 1964 | 2367 | 3.229338 | 25.62296 | | | | | | France | 71.28 | France |
| 6 | France | 1965 | 2526 | 2.71474 | 25.7495 | 25.8 | | | | | France | 71.1 | France |
| 6 | France | 1966 | 2728 | 2.570721 | 26.47594 | | | | | | France | 71.51 | France |
| 6 | France | 1967 | 2915 | 2.818319 | 26.18846 | | | | | | France | 71.5 | France |
| 6 | France | 1968 | 3163 | 4.552129 | 26.57132 | | | | | | France | 71.49 | France |
| 6 | France | 1969 | 3531 | 6.039326 | 28.73958 | | | | | | France | 71.21 | France |
| 6 | France | 1970 | 3857 | 5.845475 | 31.08583 | 26 | | | | | France | 72.1 | France |
| 6 | France | 1971 | 4203 | 5.397514 | 31.76096 | | | | | 38.64927 | France | 72.07 | France |
| 6 | France | 1972 | 4535 | 6.063004 | 32.39192 | | | | | 38.6979 | France | 72.32 | France |
| 6 | France | 1973 | 5008 | 7.380597 | 34.27934 | | | | | 37.94067 | France | 72.47 | France |
| 6 | France | 1974 | 5594 | 13.64932 | 42.39096 | | | | | 37.56777 | France | 72.81 | France |
| 6 | France | 1975 | 6077 | 11.68593 | 36.92751 | 26.8 | | | | 37.66209 | France | 72.9 | France |
| 6 | France | 1976 | 6673 | 9.625493 | 39.89237 | | | | | 37.49077 | France | 73.14 | France |
| 6 | France | 1977 | 7292 | 9.494556 | 40.84986 | | | | | 36.88102 | France | 73.73 | France |
| 6 | France | 1978 | 8031 | 9.250559 | 39.48853 | | | | | 36.23006 | France | 73.85 | France |
| 6 | France | 1979 | 8948 | 10.64674 | 41.87905 | | | | | 35.84073 | France | 74.13 | France |
| 6 | France | 1980 | 9891 | 13.53911 | 44.26932 | 34.5 | 0.27806 | 0.3287 | 27.236 | 36.0303 | France | 74.24 | France |
| 6 | France | 1981 | 10929 | 13.33333 | 46.10194 | | | | | 35.17299 | France | 74.4 | France |
| 6 | France | 1982 | 11869 | 11.97848 | 45.5013 | | | | | 34.09864 | France | 74.77 | France |
| 6 | France | 1983 | 12518 | 9.459548 | 45.12784 | | | | | 33.51458 | France | 74.74 | France |
| 6 | France | 1984 | 13145 | 7.673803 | 47.64643 | | | | | 33.12116 | France | 75.23 | France |
| 6 | France | 1985 | 13746 | 5.831099 | 47.15918 | 36.3 | | | | 32.78713 | France | 75.31 | France |
| 6 | France | 1986 | 14308 | 2.538527 | 41.34467 | | | | | 31.80395 | France | 75.58 | France |
| 6 | France | 1987 | 14940 | 3.288898 | 41.14437 | | | | | 30.94018 | France | 76.15 | France |
| 6 | France | 1988 | 16040 | 2.700817 | 42.52645 | | | | | 30.74395 | France | 76.4 | France |
| 6 | France | 1989 | 17193 | 3.498302 | 45.68644 | | | | | 30.22196 | France | 76.55 | France |
| 6 | France | 1990 | 18244 | 3.380057 | 45.13057 | 36.9 | | | | 29.69998 | France | 76.84 | France |
| 6 | France | 1991 | 19021 | 3.216935 | 45.04747 | | | | | 29.52016 | France | 77.02 | France |
| 6 | France | 1992 | 19746 | 2.365765 | 44.02612 | | | | | 29.15713 | France | 77.3 | France |
| 6 | France | 1993 | 19920 | 2.10601 | 41.84121 | | | | | 27.45125 | France | 77.36 | France |
| 6 | France | 1994 | 20695 | 1.660873 | 43.40138 | | | | | 27.18095 | France | 77.78 | France |
| 6 | France | 1995 | 21545 | 1.778116 | 44.69141 | 37.3 | | | | 27.2759 | France | 77.88 | France |
| 6 | France | 1996 | 22288 | 2.008362 | 45.60446 | | | | | 26.60661 | France | 78.07 | France |
| 6 | France | 1997 | 23370 | 1.200322 | 49.3604 | | | | | 26.04123 | France | 78.43 | France |
| 6 | France | 1998 | 24412 | 0.672597 | | | | | | 25.94564 | France | 78.6 | France |
| 6 | France | 1999 | 25111 | 0.5 | | | | | | 25.49264 | France | 78.76 | France |
| 6 | France | 2000 | 26690 | 1.691542 | | 37.3 | | | | 25.48119 | France | 79.06 | France |
| 6 | France | 2001 | 28043 | 1.663405 | | | | | | 25.5017 | France | 79.23 | France |
| 6 | France | 2002 | 28829 | 1.924928 | | | | | | 25.30812 | France | 79.43 | France |
| 6 | France | 2003 | 29210 | 2.101039 | | | | | | 24.47357 | France | 79.43 | France |
| 7 | West Gerr | 1960 | 2284 | | 35.45968 | 61 | | | | 44.98724 | Germany | 69.3 | West Germany |
| 7 | West Gerr | 1961 | 2388 | 2.515723 | 33.83678 | | | | | | Germany | 69.82 | West Germany |
| 7 | West Gerr | 1962 | 2527 | 2.760736 | 33.46638 | | | | | | Germany | 70.06 | West Germany |
| 7 | West Gerr | 1963 | 2610 | 2.985075 | 34.16063 | | | | | | Germany | 70.01 | West Germany |
| 7 | West Gerr | 1964 | 2806 | 2.318841 | 34.56614 | | | | | | Germany | 70.69 | West Germany |
| 7 | West Gerr | 1965 | 3005 | 3.399433 | 35.8168 | 58.9 | | | | | Germany | 70.63 | West Germany |
| 7 | West Gerr | 1966 | 3168 | 3.561644 | 36.62823 | | | | | | Germany | 70.67 | West Germany |
| 7 | West Gerr | 1967 | 3241 | 1.587302 | 37.22666 | | | | | | Germany | 70.91 | West Germany |
| 7 | West Gerr | 1968 | 3571 | 1.041667 | 39.09016 | | | | | | Germany | 70.56 | West Germany |
| 7 | West Gerr | 1969 | 3998 | 2.061856 | 40.57124 | | | | | | Germany | 70.39 | West Germany |
| 7 | West Gerr | 1970 | 4367 | 3.282828 | 40.31838 | 57 | | | | 38.21701 | Germany | 70.63 | West Germany |
| 7 | West Gerr | 1971 | 4686 | 5.134474 | 39.73991 | | | | | 48.00126 | Germany | 70.8 | West Germany |
| 7 | West Gerr | 1972 | 5055 | 5.348837 | 39.23 | | | | | 46.94689 | Germany | 71.04 | West Germany |
| 7 | West Gerr | 1973 | 5553 | 6.843267 | 40.75988 | | | | | 46.53436 | Germany | 71.27 | West Germany |
| 7 | West Gerr | 1974 | 6074 | 6.818182 | 48.39369 | | | | | 45.70887 | Germany | 71.62 | West Germany |
| 7 | West Gerr | 1975 | 6603 | 5.996131 | 46.45783 | 46.8 | | | | 43.37009 | Germany | 71.53 | West Germany |
| 7 | West Gerr | 1976 | 7367 | 4.379562 | 49.10308 | | | | | 43.61361 | Germany | 72.03 | West Germany |
| 7 | West Gerr | 1977 | 8090 | 3.496504 | 48.56479 | | | | | 43.16602 | Germany | 72.72 | West Germany |
| 7 | West Gerr | 1978 | 8928 | 2.533784 | 47.07179 | | | | | 42.96508 | Germany | 72.73 | West Germany |
| 7 | West Gerr | 1979 | 10067 | 3.789127 | 49.48575 | | | | | 43.08099 | Germany | 73.19 | West Germany |
| 7 | West Gerr | 1980 | 11083 | 5.238095 | 53.33619 | 52.5 | 0.33738 | 0.32564 | 27.018 | 36.04203 | Germany | 73.44 | West Germany |
| 7 | West Gerr | 1981 | 12115 | 6.334842 | 56.67342 | | | | | 35.42349 | Germany | 73.67 | West Germany |
| 7 | West Gerr | 1982 | 12761 | 5.390071 | 57.34184 | | | | | 34.77847 | Germany | 74 | West Germany |
| 7 | West Gerr | 1983 | 13519 | 3.230148 | 55.48264 | | | | | 35.08514 | Germany | 74.26 | West Germany |
| 7 | West Gerr | 1984 | 14481 | 2.346806 | 58.79181 | | | | | 34.51585 | Germany | 74.84 | West Germany |
| 7 | West Gerr | 1985 | 15291 | 2.038217 | 61.51943 | 58.5 | | | | 34.85139 | Germany | 75 | West Germany |
| 7 | West Gerr | 1986 | 15998 | -0.24969 | 55.11741 | | | | | 34.36679 | Germany | 75.25 | West Germany |
| 7 | West Gerr | 1987 | 16679 | 0.125156 | 52.91488 | | | | | 34.09019 | Germany | 75.66 | West Germany |
| 7 | West Gerr | 1988 | 17786 | 1.125 | 53.91034 | | | | | 33.77234 | Germany | 75.88 | West Germany |
| 7 | West Gerr | 1989 | 18994 | 2.843016 | 57.66485 | | | | | 33.96273 | Germany | 76 | West Germany |
| 7 | West Gerr | 1990 | 20465 | 2.764423 | 58.3615 | 56.2 | | | | | Germany | 76 | West Germany |
| 7 | West Gerr | 1991 | 21602 | 3.74269 | | | | | | | Germany | 76.18 | West Germany |
| 7 | West Gerr | 1992 | 22154 | 4.058625 | | | | | | | Germany | 76.56 | West Germany |
| 7 | West Gerr | 1993 | 21878 | 3.68364 | | | | | | | Germany | 76.54 | West Germany |
| 7 | West Gerr | 1994 | 22371 | 2.821317 | | | | | | | Germany | 76.83 | West Germany |
| 7 | West Gerr | 1995 | 23035 | 1.626016 | | | | | | | Germany | 76.94 | West Germany |
| 7 | West Gerr | 1996 | 23742 | 1.3 | | | | | | | Germany | 77.11 | West Germany |
| 7 | West Gerr | 1997 | 24156 | 1.7769 | | | | | | | Germany | 77.49 | West Germany |
| 7 | West Gerr | 1998 | 24931 | 0.872939 | | | | | | | Germany | 77.8 | West Germany |
| 7 | West Gerr | 1999 | 25755 | 0.673077 | | | | | | | Germany | 77.98 | West Germany |
| 7 | West Gerr | 2000 | 26943 | | | | | | | | Germany | 78.24 | West Germany |
| 7 | West Gerr | 2001 | 27449 | | | | | | | | Germany | 78.56 | West Germany |
| 7 | West Gerr | 2002 | 28348 | | | | | | | | Germany | 78.64 | West Germany |
| 7 | West Gerr | 2003 | 28855 | | | | | | | | Germany | 78.68 | West Germany |

| | | | | | | | | | | | | | |
|---|---|---|---|---|---|---|---|---|---|---|---|---|---|
| 4 | Belgium | 1960 | 1782 | | 77.34178 | 27.1 | | | | | Belgium | 69.63 | Belgium |
| 4 | Belgium | 1961 | 1883 | 0.983146 | 80.01627 | | | | | | Belgium | 70.5 | Belgium |
| 4 | Belgium | 1962 | 2018 | 1.404729 | 82.43776 | | | | | | Belgium | 70.23 | Belgium |
| 4 | Belgium | 1963 | 2122 | 2.15334 | 85.66539 | | | | | | Belgium | 70.04 | Belgium |
| 4 | Belgium | 1964 | 2288 | 4.175618 | 86.62045 | | | | | | Belgium | 70.71 | Belgium |
| 4 | Belgium | 1965 | 2414 | 4.059802 | 85.26191 | 28.5 | | | | | Belgium | 70.56 | Belgium |
| 4 | Belgium | 1966 | 2559 | 4.173892 | 89.37012 | | | | | | Belgium | 70.63 | Belgium |
| 4 | Belgium | 1967 | 2719 | 2.912852 | 86.23965 | | | | | | Belgium | 70.92 | Belgium |
| 4 | Belgium | 1968 | 2958 | 2.703327 | 90.62302 | | | | | | Belgium | 70.61 | Belgium |
| 4 | Belgium | 1969 | 3308 | 3.745782 | 97.94964 | | | | | | Belgium | 70.68 | Belgium |
| 4 | Belgium | 1970 | 3797 | 3.914128 | 100.4281 | 28.7 | | | | | Belgium | 70.95 | Belgium |
| 4 | Belgium | 1971 | 4129 | 4.340568 | 97.9751 | | | | | 42.50227 | Belgium | 71.08 | Belgium |
| 4 | Belgium | 1972 | 4512 | 5.4475 | 97.54892 | | | | | 41.94415 | Belgium | 71.42 | Belgium |
| 4 | Belgium | 1973 | 5039 | 6.955278 | 107.7751 | | | | | 42.51509 | Belgium | 71.63 | Belgium |
| 4 | Belgium | 1974 | 5707 | 12.67789 | 120.5175 | | | | | 43.29301 | Belgium | 71.98 | Belgium |
| 4 | Belgium | 1975 | 6146 | 12.7682 | 104.7795 | 30.3 | | | | 40.01701 | Belgium | 71.97 | Belgium |
| 4 | Belgium | 1976 | 6854 | 9.1587 | 110.8381 | | | | | 40.13395 | Belgium | 72.12 | Belgium |
| 4 | Belgium | 1977 | 7326 | 7.110848 | 109.5376 | | | | | 39.49528 | Belgium | 72.76 | Belgium |
| 4 | Belgium | 1978 | 8056 | 4.470595 | 105.6856 | | | | | 38.45702 | Belgium | 72.71 | Belgium |
| 4 | Belgium | 1979 | 8921 | 4.469084 | 116.2898 | | | | | 38.20924 | Belgium | 73.21 | Belgium |
| 4 | Belgium | 1980 | 10156 | 6.650975 | 117.1688 | 31.6 | 0.26864 | 0.28246 | 21.876 | 36.84375 | Belgium | 73.25 | Belgium |
| 4 | Belgium | 1981 | 11079 | 7.628274 | 126.3526 | | | | | 34.46517 | Belgium | 73.66 | Belgium |
| 4 | Belgium | 1982 | 11827 | 8.725851 | 134.4237 | | | | | 34.4062 | Belgium | 73.88 | Belgium |
| 4 | Belgium | 1983 | 12334 | 7.66337 | 137.1891 | | | | | 34.14059 | Belgium | 73.89 | Belgium |
| 4 | Belgium | 1984 | 13113 | 6.347517 | 146.6429 | | | | | 33.48085 | Belgium | 74.37 | Belgium |
| 4 | Belgium | 1985 | 13735 | 4.867676 | 140.5551 | 32.6 | | | | 34.2135 | Belgium | 74.48 | Belgium |
| 4 | Belgium | 1986 | 14292 | 1.295501 | 126.711 | | | | | 33.22915 | Belgium | 74.68 | Belgium |
| 4 | Belgium | 1987 | 15013 | 1.554483 | 122.6466 | | | | | 32.1018 | Belgium | 75.29 | Belgium |
| 4 | Belgium | 1988 | 16209 | 1.162051 | 129.2056 | | | | | 32.88089 | Belgium | 75.6 | Belgium |
| 4 | Belgium | 1989 | 17345 | 3.10545 | 138.3847 | | | | | 33.22943 | Belgium | 75.66 | Belgium |
| 4 | Belgium | 1990 | 18526 | 3.452823 | 134.0066 | 32.6 | | | | 32.6643 | Belgium | 76.07 | Belgium |
| 4 | Belgium | 1991 | 19453 | 3.208552 | 130.8264 | | | | | 30.66162 | Belgium | 76.21 | Belgium |
| 4 | Belgium | 1992 | 20123 | 2.429914 | 126.436 | | | | | 30.11093 | Belgium | 76.37 | Belgium |
| 4 | Belgium | 1993 | 20308 | 2.753963 | 120.238 | | | | | 28.99867 | Belgium | 76.38 | Belgium |
| 4 | Belgium | 1994 | 21340 | 2.377799 | 125.5379 | | | | | 28.92364 | Belgium | 76.72 | Belgium |
| 4 | Belgium | 1995 | 22248 | 1.466814 | 129.3607 | 30.5 | | | | 29.53443 | Belgium | 76.85 | Belgium |
| 4 | Belgium | 1996 | 22687 | 2.058919 | 131.875 | | | | | 29.14131 | Belgium | 77.2 | Belgium |
| 4 | Belgium | 1997 | 23416 | 1.627459 | 141.3144 | | | | | 29.25781 | Belgium | 77.41 | Belgium |
| 4 | Belgium | 1998 | 24164 | 0.953623 | 149.6824 | | | | | 28.63953 | Belgium | 77.49 | Belgium |
| 4 | Belgium | 1999 | 24792 | 1.119242 | | | | | | 27.95392 | Belgium | 77.66 | Belgium |
| 4 | Belgium | 2000 | 26631 | 2.546246 | | 28 | | | | 28.08952 | Belgium | 77.78 | Belgium |
| 4 | Belgium | 2001 | 28001 | 2.471273 | | | | | | 27.25738 | Belgium | 78.09 | Belgium |
| 4 | Belgium | 2002 | 29330 | 1.643454 | | | | | | 27.01092 | Belgium | 78.18 | Belgium |
| 4 | Belgium | 2003 | 30082 | 1.591312 | | | | | | 26.48478 | Belgium | 78.34 | Belgium |
| 5 | Denmark | 1960 | 2329 | | 65.7822 | 46.1 | | | | | Denmark | 72.2 | Denmark |
| 5 | Denmark | 1961 | 2485 | 3.451629 | 61.54067 | | | | | | Denmark | 72.45 | Denmark |
| 5 | Denmark | 1962 | 2665 | 7.37782 | 60.33738 | | | | | | Denmark | 72.34 | Denmark |
| 5 | Denmark | 1963 | 2700 | 6.105033 | 60.41604 | | | | | | Denmark | 72.42 | Denmark |
| 5 | Denmark | 1964 | 2980 | 3.092783 | 61.6781 | | | | | | Denmark | 72.51 | Denmark |
| 5 | Denmark | 1965 | 3177 | 5.45 | 61.38874 | 45.5 | | | | | Denmark | 72.39 | Denmark |
| 5 | Denmark | 1966 | 3377 | 7.06496 | 59.87274 | | | | | | Denmark | 72.46 | Denmark |
| 5 | Denmark | 1967 | 3580 | 8.207853 | 57.85473 | | | | | | Denmark | 72.94 | Denmark |
| 5 | Denmark | 1968 | 3892 | 7.953615 | 57.87989 | | | | | | Denmark | 73.15 | Denmark |
| 5 | Denmark | 1969 | 4352 | 3.487931 | 58.46833 | | | | | | Denmark | 73.24 | Denmark |
| 5 | Denmark | 1970 | 4541 | 6.514837 | 60.32181 | 44.5 | | | | | Denmark | 73.37 | Denmark |
| 5 | Denmark | 1971 | 4858 | 5.869877 | 58.49475 | | | | | 31.78034 | Denmark | 73.43 | Denmark |
| 5 | Denmark | 1972 | 5262 | 6.562348 | 54.94262 | | | | | 31.53147 | Denmark | 73.45 | Denmark |
| 5 | Denmark | 1973 | 5719 | 9.303389 | 60.4651 | | | | | 30.28473 | Denmark | 73.69 | Denmark |
| 5 | Denmark | 1974 | 6121 | 15.2752 | 68.13898 | | | | | 29.58704 | Denmark | 73.82 | Denmark |
| 5 | Denmark | 1975 | 6565 | 9.605614 | 62.65065 | 44.9 | | | | 29.25056 | Denmark | 74.09 | Denmark |
| 5 | Denmark | 1976 | 7371 | 9 | 63.9493 | | | | | 28.24987 | Denmark | 73.76 | Denmark |
| 5 | Denmark | 1977 | 7901 | 11.13914 | 62.84295 | | | | | 28.02203 | Denmark | 74.66 | Denmark |
| 5 | Denmark | 1978 | 8585 | 10.00894 | 59.20886 | | | | | 27.56307 | Denmark | 74.42 | Denmark |
| 5 | Denmark | 1979 | 9566 | 9.611055 | 62.88595 | | | | | 26.88064 | Denmark | 74.24 | Denmark |
| 5 | Denmark | 1980 | 10362 | 12.30532 | 68.16064 | 43.4 | 0.29344 | 0.33284 | 22.218 | 28.23314 | Denmark | 74.13 | Denmark |
| 5 | Denmark | 1981 | 11106 | 11.79159 | 74.18737 | | | | | 26.5771 | Denmark | 74.25 | Denmark |
| 5 | Denmark | 1982 | 12115 | 10.08994 | 74.13429 | | | | | 26.75725 | Denmark | 74.59 | Denmark |
| 5 | Denmark | 1983 | 12822 | 6.907309 | 72.48873 | | | | | 26.98976 | Denmark | 74.46 | Denmark |
| 5 | Denmark | 1984 | 13777 | 6.322079 | 73.97858 | | | | | 27.29058 | Denmark | 74.61 | Denmark |
| 5 | Denmark | 1985 | 14698 | 4.652379 | 74.85047 | 42.5 | | | | 27.62063 | Denmark | 74.48 | Denmark |
| 5 | Denmark | 1986 | 15608 | 3.721279 | 66.16891 | | | | | 28.24608 | Denmark | 74.63 | Denmark |
| 5 | Denmark | 1987 | 16024 | 3.985071 | 62.58148 | | | | | 27.91802 | Denmark | 74.74 | Denmark |
| 5 | Denmark | 1988 | 16766 | 4.550191 | 63.53234 | | | | | 27.08609 | Denmark | 74.84 | Denmark |
| 5 | Denmark | 1989 | 17418 | 4.784053 | 67.06669 | | | | | 27.02068 | Denmark | 74.86 | Denmark |
| 5 | Denmark | 1990 | 18237 | 2.652716 | 66.58468 | 49.9 | | | | 26.54429 | Denmark | 74.86 | Denmark |
| 5 | Denmark | 1991 | 19070 | 2.36796 | 68.53211 | | | | | 26.16956 | Denmark | 75.23 | Denmark |
| 5 | Denmark | 1992 | 19829 | 2.101981 | 66.43089 | | | | | 26.34534 | Denmark | 75.26 | Denmark |
| 5 | Denmark | 1993 | 20199 | 1.250985 | 63.97609 | | | | | 25.19012 | Denmark | 75.18 | Denmark |
| 5 | Denmark | 1994 | 21691 | 1.994357 | 65.61267 | | | | | 25.31154 | Denmark | 75.44 | Denmark |
| 5 | Denmark | 1995 | 22693 | 2.098436 | 66.71786 | 47 | | | | 25.75751 | Denmark | 75.28 | Denmark |
| 5 | Denmark | 1996 | 23781 | 2.11136 | 66.07381 | | | | | 25.95113 | Denmark | 75.66 | Denmark |
| 5 | Denmark | 1997 | 24878 | 2.195791 | 68.58707 | | | | | 26.04515 | Denmark | 76.03 | Denmark |
| 5 | Denmark | 1998 | 25669 | 1.853178 | | | | | | 26.00064 | Denmark | 76.44 | Denmark |
| 5 | Denmark | 1999 | 27113 | 2.478685 | | | | | | 26.2223 | Denmark | 76.58 | Denmark |
| 5 | Denmark | 2000 | 28798 | 2.924779 | | 46.5 | | | | 27.18491 | Denmark | 76.81 | Denmark |
| 5 | Denmark | 2001 | 29837 | 2.35 | | | | | | 26.28203 | Denmark | 76.97 | Denmark |
| 5 | Denmark | 2002 | 30318 | 2.426315 | | | | | | 26.40067 | Denmark | 77.09 | Denmark |
| 5 | Denmark | 2003 | 30853 | 2.09062 | | | | | | 26.40539 | Denmark | 77.69 | Denmark |

| | | | | | | | | | | | | | |
|---|---|---|---|---|---|---|---|---|---|---|---|---|---|
| 20 | Australia | 1960 | 2373 | | 31.26644 | 50.4 | | | | | Australia | 70.87 | Australia |
| 20 | Australia | 1961 | 2346 | 2.512132 | 29.82107 | | | | | | Australia | 71.13 | Australia |
| 20 | Australia | 1962 | 2539 | -0.27847 | 30.15854 | | | | | | Australia | 70.9 | Australia |
| 20 | Australia | 1963 | 2717 | 0.530578 | 32.0554 | | | | | | Australia | 70.96 | Australia |
| 20 | Australia | 1964 | 2873 | 2.361111 | 31.52728 | | | | | | Australia | 70.57 | Australia |
| 20 | Australia | 1965 | 2973 | 3.962008 | 31.0356 | 48.9 | | | | | Australia | 70.8 | Australia |
| 20 | Australia | 1966 | 3230 | 2.975724 | 29.97315 | | | | | | Australia | 70.68 | Australia |
| 20 | Australia | 1967 | 3404 | 3.193916 | 30.11419 | | | | | | Australia | 71.04 | Australia |
| 20 | Australia | 1968 | 3788 | 2.652911 | 28.34859 | | | | | | Australia | 70.69 | Australia |
| 20 | Australia | 1969 | 4162 | 2.919359 | 29.82114 | | | | | | Australia | 71.06 | Australia |
| 20 | Australia | 1970 | 4490 | 3.906068 | 28.96243 | 48.3 | | | | | Australia | 70.66 | Australia |
| 20 | Australia | 1971 | 4677 | 6.063996 | 27.80747 | | | | | 37.29359 | Australia | 71.43 | Australia |
| 20 | Australia | 1972 | 5013 | 5.864979 | 27.70299 | | | | | 36.80088 | Australia | 71.77 | Australia |
| 20 | Australia | 1973 | 5595 | 9.465922 | 29.4404 | | | | | 35.43585 | Australia | 72.06 | Australia |
| 20 | Australia | 1974 | 6024 | 15.11014 | 31.63861 | | | | | 36.88676 | Australia | 71.85 | Australia |
| 20 | Australia | 1975 | 6683 | 15.07196 | 28.90041 | 47 | | | | 37.10455 | Australia | 72.78 | Australia |
| 20 | Australia | 1976 | 7140 | 13.52391 | 31.20762 | | | | | 36.66529 | Australia | 72.79 | Australia |
| 20 | Australia | 1977 | 7482 | 12.30024 | 30.8631 | | | | | 35.99936 | Australia | 73.47 | Australia |
| 20 | Australia | 1978 | 8272 | 7.923674 | 32.15132 | | | | | 35.65282 | Australia | 73.74 | Australia |
| 20 | Australia | 1979 | 9174 | 9.090001 | 34.95831 | | | | | 35.81057 | Australia | 74.33 | Australia |
| 20 | Australia | 1980 | 10203 | 10.12658 | 33.86125 | 47.5 | 0.3173 | 0.31798 | 29.696 | 37.0267 | Australia | 74.57 | Australia |
| 20 | Australia | 1981 | 11513 | 9.691745 | 33.17373 | | | | | 35.92434 | Australia | 74.78 | Australia |
| 20 | Australia | 1982 | 11537 | 11.14551 | 31.65774 | | | | | 34.74367 | Australia | 74.72 | Australia |
| 20 | Australia | 1983 | 12300 | 10.11356 | 30.67814 | | | | | 34.8257 | Australia | 75.44 | Australia |
| 20 | Australia | 1984 | 13120 | 3.950185 | 34.641 | | | | | 35.29966 | Australia | 75.51 | Australia |
| 20 | Australia | 1985 | 14019 | 6.739049 | 35.24625 | 48.4 | | | | 35.07288 | Australia | 75.43 | Australia |
| 20 | Australia | 1986 | 14537 | 9.084532 | 34.7444 | | | | | 32.77786 | Australia | 76.07 | Australia |
| 20 | Australia | 1987 | 15554 | 8.488746 | 34.72344 | | | | | 31.35881 | Australia | 76.08 | Australia |
| 20 | Australia | 1988 | 16524 | 7.231772 | 34.08369 | | | | | 29.87518 | Australia | 76.35 | Australia |
| 20 | Australia | 1989 | 17255 | 7.559425 | 34.35854 | | | | | 29.96091 | Australia | 76.2 | Australia |
| 20 | Australia | 1990 | 17322 | 7.27226 | 34.46177 | 48.4 | | | | 28.9668 | Australia | 77.04 | Australia |
| 20 | Australia | 1991 | 17652 | 3.22268 | 35.23764 | | | | | 28.168 | Australia | 77.48 | Australia |
| 20 | Australia | 1992 | 18496 | 0.985915 | 37.69257 | | | | | 27.97166 | Australia | 77.47 | Australia |
| 20 | Australia | 1993 | 19432 | 1.81311 | 38.35179 | | | | | 28.00918 | Australia | 78.01 | Australia |
| 20 | Australia | 1994 | 20583 | 1.894977 | 39.71843 | | | | | 28.13992 | Australia | 77.85 | Australia |
| 20 | Australia | 1995 | 21773 | 4.638136 | 40.15326 | 48.6 | | | | 27.82765 | Australia | 78.26 | Australia |
| 20 | Australia | 1996 | 22562 | 2.61242 | 39.97738 | | | | | 27.10232 | Australia | 78.35 | Australia |
| 20 | Australia | 1997 | 23649 | 0.250417 | 42.1768 | | | | | 27.07669 | Australia | 78.63 | Australia |
| 20 | Australia | 1998 | 24853 | 0.853455 | 41.36496 | | | | | 26.28313 | Australia | 79.03 | Australia |
| 20 | Australia | 1999 | 26283 | 1.465428 | | | | | | 26.19073 | Australia | 79.29 | Australia |
| 20 | Australia | 2000 | 27403 | 4.475183 | | 43.6 | | | | 25.56668 | Australia | 79.63 | Australia |
| 20 | Australia | 2001 | 28492 | 4.380841 | | | | | | 25.4391 | Australia | 80.03 | Australia |
| 20 | Australia | 2002 | 29819 | 3.003171 | | | | | | 25.94952 | Australia | 80.07 | Australia |
| 20 | Australia | 2003 | 31273 | 2.770735 | | | | | | | Australia | 80.47 | Australia |
| 3 | Austria | 1960 | 1796 | | 48.12609 | 48.7 | | | | | Austria | 68.75 | Austria |
| 3 | Austria | 1961 | 1899 | 3.542256 | 46.52596 | | | | | | Austria | 69.72 | Austria |
| 3 | Austria | 1962 | 1977 | 4.381785 | 47.17101 | | | | | | Austria | 69.5 | Austria |
| 3 | Austria | 1963 | 2074 | 2.70877 | 47.97725 | | | | | | Austria | 69.64 | Austria |
| 3 | Austria | 1964 | 2224 | 3.868571 | 48.4151 | | | | | | Austria | 70.13 | Austria |
| 3 | Austria | 1965 | 2336 | 4.930911 | 49.6133 | 47.6 | | | | | Austria | 69.93 | Austria |
| 3 | Austria | 1966 | 2537 | 2.205882 | 50.31641 | | | | | | Austria | 70.24 | Austria |
| 3 | Austria | 1967 | 2668 | 3.975 | 49.68385 | | | | | | Austria | 70.12 | Austria |
| 3 | Austria | 1968 | 2908 | 2.773103 | 50.41092 | | | | | | Austria | 70.27 | Austria |
| 3 | Austria | 1969 | 3239 | 3.080402 | 54.23251 | | | | | | Austria | 70.04 | Austria |
| 3 | Austria | 1970 | 3732 | 4.372825 | 59.71196 | 46.5 | | | | | Austria | 70.09 | Austria |
| 3 | Austria | 1971 | 4100 | 4.704262 | 59.18537 | | | | | 41.21851 | Austria | 70.3 | Austria |
| 3 | Austria | 1972 | 4513 | 6.362063 | 59.09092 | | | | | 41.52563 | Austria | 70.62 | Austria |
| 3 | Austria | 1973 | 4969 | 7.524082 | 59.26243 | | | | | 42.23682 | Austria | 71.2 | Austria |
| 3 | Austria | 1974 | 5622 | 9.521791 | 64.81173 | | | | | 42.26027 | Austria | 71.18 | Austria |
| 3 | Austria | 1975 | 6147 | 8.445255 | 61.59815 | 45.6 | | | | 40.12814 | Austria | 71.32 | Austria |
| 3 | Austria | 1976 | 6810 | 7.318689 | 65.17087 | | | | | 39.3979 | Austria | 71.8 | Austria |
| 3 | Austria | 1977 | 7588 | 5.475 | 65.20969 | | | | | 39.05883 | Austria | 72.15 | Austria |
| 3 | Austria | 1978 | 8117 | 3.579047 | 64.74067 | | | | | 39.23565 | Austria | 72.21 | Austria |
| 3 | Austria | 1979 | 9288 | 3.707094 | 69.69872 | | | | | 38.1772 | Austria | 72.55 | Austria |
| 3 | Austria | 1980 | 10312 | 6.32539 | 73.99586 | 61.8 | 0.26338 | 0.31104 | 26.642 | 38.09647 | Austria | 72.69 | Austria |
| 3 | Austria | 1981 | 11242 | 6.806862 | 76.07967 | | | | | 37.83085 | Austria | 72.99 | Austria |
| 3 | Austria | 1982 | 12148 | 5.440414 | 71.68456 | | | | | 36.90459 | Austria | 73.15 | Austria |
| 3 | Austria | 1983 | 13048 | 3.335381 | 70.53411 | | | | | 36.41043 | Austria | 73.22 | Austria |
| 3 | Austria | 1984 | 13533 | 5.664864 | 75.5127 | | | | | 35.23595 | Austria | 73.77 | Austria |
| 3 | Austria | 1985 | 14296 | 3.189694 | 79.06573 | 60 | | | | 35.58244 | Austria | 73.99 | Austria |
| 3 | Austria | 1986 | 14921 | 1.700921 | 70.92529 | | | | | 35.29153 | Austria | 74.46 | Austria |
| 3 | Austria | 1987 | 15549 | 1.4 | 69.634 | | | | | 34.79196 | Austria | 74.91 | Austria |
| 3 | Austria | 1988 | 16595 | 1.931295 | 74.93547 | | | | | 34.58339 | Austria | 75.38 | Austria |
| 3 | Austria | 1989 | 17768 | 2.563896 | 78.90685 | | | | | 34.11145 | Austria | 75.47 | Austria |
| 3 | Austria | 1990 | 19070 | 3.262322 | 79.02966 | 57.9 | | | | 34.11053 | Austria | 75.74 | Austria |
| 3 | Austria | 1991 | 20172 | 3.334348 | 78.7691 | | | | | 33.98061 | Austria | 75.8 | Austria |
| 3 | Austria | 1992 | 20960 | 4.029763 | 76.00291 | | | | | 33.03325 | Austria | 76 | Austria |
| 3 | Austria | 1993 | 21220 | 3.625806 | 73.35833 | | | | | 32.18623 | Austria | 76.23 | Austria |
| 3 | Austria | 1994 | 22139 | 2.959065 | 75.14964 | | | | | 32.13828 | Austria | 76.55 | Austria |
| 3 | Austria | 1995 | 22976 | 2.2501 | 77.78494 | 55.7 | | | | 32.44965 | Austria | 76.78 | Austria |
| 3 | Austria | 1996 | 24008 | 1.843557 | 80.74392 | | | | | 32.29562 | Austria | 77.04 | Austria |
| 3 | Austria | 1997 | 24472 | 1.325 | 85.25793 | | | | | 32.72698 | Austria | 77.43 | Austria |
| 3 | Austria | 1998 | 25314 | 0.90468 | | | | | | 32.84259 | Austria | 77.79 | Austria |
| 3 | Austria | 1999 | 26558 | 0.562393 | | | | | | 32.90003 | Austria | 77.97 | Austria |
| 3 | Austria | 2000 | 28359 | 2.350462 | | 55 | | | | 32.77485 | Austria | 78.24 | Austria |
| 3 | Austria | 2001 | 28855 | 2.659962 | | | | | | 32.1686 | Austria | 78.73 | Austria |
| 3 | Austria | 2002 | 29942 | 1.810212 | | | | | | 31.98478 | Austria | 78.86 | Austria |
| 3 | Austria | 2003 | 30796 | 1.363419 | | | | | | 31.73713 | Austria | 78.86 | Austria |
